# Post‐prostatic‐massage urine exosomes of men with chronic prostatitis/chronic pelvic pain syndrome carry prostate‐cancer‐typical microRNAs and activate proto‐oncogenes

**DOI:** 10.1002/1878-0261.13329

**Published:** 2022-11-17

**Authors:** Laura Schneider, Temuujin Dansranjav, Elena Neumann, Hang Yan, Adrian Pilatz, Hans‐Christian Schuppe, Florian Wagenlehner, Undraga Schagdarsurengin

**Affiliations:** ^1^ Clinic of Urology, Pediatric Urology and Andrology Justus‐Liebig‐University Giessen Germany; ^2^ Working Group “Epigenetics of the Urogenital System,” Clinic of Urology, Pediatric Urology and Andrology Justus‐Liebig‐University Giessen Germany; ^3^ Department of Rheumatology and Clinical Immunology Campus Kerckhoff, Justus‐Liebig‐University of Giessen Bad Nauheim Germany

**Keywords:** biomarker, CP/CPPS, exosomes, liquid biopsies, microRNA, prostate cancer

## Abstract

Chronic prostatitis/chronic pelvic pain syndrome (CP/CPPS) has a high prevalence of up to 15% and accounts for 90–95% of prostatitis diagnoses, and yet its etiopathogenesis and link to prostate cancer (PCa) are still unclear. Here, we investigated microRNAs in exosomes isolated from blood and post‐prostatic‐massage urine of CP/CPPS type IIIb patients and healthy men. THP‐1 monocytes (human leukemia monocytic cell line) were treated with exosomes and subjected to mRNA arrays “Cancer Inflammation and Immunity Crosstalk” and “Transcription Factors.” Using The Cancer Genome Atlas, the expression of CP/CPPS‐associated microRNAs was analyzed in PCa and normal prostate tissue. *In silico* functional studies were carried out to explore the disease ontology of CP/CPPS. In CP/CPPS, urine exosomes exhibited significant upregulation of eight PCa‐specific microRNAs (e.g., hsa‐miR‐501, hsa‐miR‐20a, and hsa‐miR‐106), whose target genes were significantly enriched for GO terms, hallmark gene sets, and pathways specific for carcinogenesis. In THP‐1 monocytes, CP/CPPS‐derived urine exosomes induced upregulation of PCa‐associated proinflammatory genes (e.g., *CCR2* and *TLR2*) and proto‐oncogene transcription factors (e.g., *MYB* and *JUNB*). In contrast, CP/CPPS‐derived blood exosomes exhibited molecular properties similar to those of healthy men. Thus, CP/CPPS exhibits molecular changes that constitute a risk for PCa and should be considered in the development of PCa biomarkers and cancer screening programs.

## Graphical abstract

Chronic prostatitis/chronic pelvic pain syndrome (CP/CPPS) has a high incidence. However, its risk for prostate cancer (PCa) is unexplored. Findings: exosomes in post‐prostatic‐massage urine of CP/CPPS patients exhibited Pca characteristic microRNA profiles and activated cancer‐associated proinflammatory and proto‐oncogene transcription factors in THP‐1 monocytes. Thus, CP/CPPS may represent a risk for PCa development and should be considered in cancer screening programs.

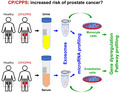

**Abbreviations**
ATF3: activating transcription factor 3
BPH: benign prostate hyperplasia
BSA: bovine serum albumin
CCL: CC‐chemokine ligand
CCR: CC‐chemokine receptor
CP/CPPS: chronic prostatitis/chronic pelvic pain syndrome
CPSI: Chronic Prostatitis Symptom Index
CRP: C‐reactive protein
DAPI: 4′,6‐diamidino‐2‐phenylindole
DAVID: Database for Annotation, Visualization and Integrated Discovery
DFG: German Research Foundation
DOSE: disease ontology semantic and enrichment
EC: endothelial cells
ECM2: endothelial cell growth medium
ELISA: enzyme‐linked immunosorbent assay
ESR: estrogen receptor
FCS: fetal calf serum
FPKM: fragments per kilo base of transcript per million mapped fragments
GAPDH: glyceraldehyde phosphate dehydrogenase
GO: gene ontology
GSEA: gene set enrichment analysis
Hsa‐miR: homo sapiens microRNA
HSP70: heat shock protein 70
HUVEC: human umbilical vein endothelial cells
ICC: immunocytochemistry
IF: immunofluorescence staining
IFN: interferon
IHC: immunohistochemistry
IL: interleukin
IPSS: International Prostate Symptom Score
KEGG: Kyoto Encyclopedia of Genes and Genomes
LPS: lipopolysaccharide
MCP‐1: monocyte chemoattractant protein‐1
MSigDB: molecular signature database
NIH: national institute of health
NOR: normal prostate tissue
PCa: prostate cancer
PMA: phorbol 12‐myristate 13‐acetate
PPM urine: post‐prostatic‐massage urine
PSA: prostate‐specific antigen
PTEN: phosphatase and tensin homolog
qPCR: quantitative polymerase chain reaction
qWB: quantitative western blot
RNA: ribonucleic acid
RPM3: Roswell Park Memorial Institute
RT: reverse transcription
SNORD44: small nucleolar RNA
STAT2: signal transducer and activator of transcription 2
TCGA: The Cancer Genome Atlas
TEM: transmission electron microscopy
TNF: tumor necrosis factor
UPOINTS: urinary, psychosocial, organ‐specific, infectious, neurological/systemic, tenderness of skeletal muscles, and sexual dysfunction

## 1 Introduction

Chronic prostatitis/chronic pelvic pain syndrome (CP/CPPS) is defined by the National Institute of Health (NIH) as genitourinary pain in the absence of uropathogenic bacteria [1]. CP/CPPS, also known as type III in the NIH classification of prostatitis syndromes, accounts for 90–95% of prostatitis diagnoses and has an estimated prevalence of up to 15% in the male population [2]. CP/CPPS is subclassified into type IIIa (inflammatory) and IIIb (non‐inflammatory) depending on the presence of leucocytes in post‐prostatic‐massage urine (further referred to as “PPM urine”) or prostatic secretion [1]. In our tertiary referral center, type IIIb accounted for 81.4% of all CP/CPPS cases [3]. However, this classification does not have implications for differential treatment [4], or does the presence of leukocytes in prostatic secretions correlate with the severity of symptoms [5] or persist in the same patient. Patients diagnosed with CP/CPPS constitute a heterogeneous group with regard to symptoms and symptom intensities and are usually treated using an individualized multimodal approach. Development of the UPOINTS system, which records the prevailing CP/CPPS symptoms (e.g., urinary, psychosocial, organ‐specific, infection, neurological, muscle tenderness, and sexual dysfunction), allowed the stratification of patients into clinical phenotypes and phenotype‐directed treatment [3, 6, 7]. The reported sickness impact of CP/CPPS is on par with that of myocardial infarction and Crohn's disease but greater than that of severe heart failure or diabetes mellitus [8]. CP/CPPS can affect men of any age (range 20–82 years) [9], and the average age for CP/CPPS occurrence among different nationalities is 47.2 years (standard deviation 15.7) [10]. Much of the etiology, pathogenesis, and long‐term health effects of CP/CPPS are still largely unknown. Possible etiological factors include non‐specific urethritis, occult infection, interstitial cystitis, cardiovascular disease, neurological disease, psychiatric conditions, and autoimmune disorders [8]. Men with CP/CPPS tend to have a 6‐fold greater history of cardiovascular disease, 5‐fold greater history of neurological disease, 2.5‐fold greater history of psychiatric disease, and 2‐fold greater history of hematopoietic, lymphatic, or infectious disease compared to men without [11].

Evidence suggests that prostatitis and prostate cancer (PCa) are closely related, but the role of the chronic inflammatory microenvironment and immune mediators in the development of PCa are not yet understood in detail [12, 13, 14, 15]. Type I and II prostatitis (acute and chronic bacterial prostatitis, respectively) are associated with PCa, whereas type III prostatitis (CP/CPPS) has not been related to the occurrence of PCa [16, 17]. Men with CP/CPPS show no clinical signs of PCa and exhibit mostly normal serum concentrations of prostate‐specific antigen (PSA). However, the possible autoimmune or autoinflammatory origin of CP/CPPS and abnormal regulation of immune cells (e.g., T‐regulatory cells and macrophages) and their related cytokines suggest that CP/CPPS may harbor the potential to trigger and/or modulate the development of PCa [2, 18, 19, 20]. Research in this direction is still scarce and complicated by the fact that prostate biopsies are not routinely performed in CP/CPPS and are not available for research. The high prevalence and morbidity of CP/CPPS, as well as the possible link to PCa upon aging, stresses the need for alternative approaches. We previously demonstrated that liquid biopsies of men with CP/CPPS are a well‐suited non‐invasive source from which profound insights can be obtained at the molecular level, providing initial hints for the potential link between CP/CPPS and PCa [21, 22, 23]. For example, in CP/CPPS, at both the systemic (i.e., in the blood) and local level (i.e., in semen), we detected epigenetic downregulation of C‐X‐C motif chemokine receptor 4 [23], which plays a critical role in establishing the tumor microenvironment in a number of malignancies, including PCa [24, 25]. In semen from patients with CP/CPPS, we also detected epigenetic downregulation of estrogen receptors (ESRs), ESR1 and ESR2, whereas estradiol concentrations were increased [22]. Frequent promoter methylation and abnormal expression of ESRs are often detected in PCa and correlate with tumor progression [26]. Moreover, CP/CPPS patients exhibit markedly decreased zinc levels in seminal plasma [21], and a reduction in zinc levels is typical for PCa and benign prostate hyperplasia (BPH) [27], which has been shown to skew the inflammatory reaction by increasing the production of IL‐1B and TNFα [28].

Exosomes are endosomal‐derived vesicles that are released by many tissue types into body fluids, including blood and urine. Exosomes contain many functional molecules, such as nucleic acids, microRNAs, and proteins, which reflect the conditional state of their tissue of origin and, therefore, are ideal candidates for the development of non‐invasive biomarkers of cancer [29]. Importantly, exosomes play a critical role in mediating cell‐to‐cell communication by transferring their cargo to targeted cells and activating different physiological processes in the acceptor cells [30]. For example, microRNAs transferred by exosomes can lead to altered gene expression in the acceptor cells and cause functional changes [31]. Analysis of exosome‐derived microRNAs has also been shown to be insightful in terms of understanding intercellular communication during the invasion of cancer cells in many tumor entities, including PCa [32].

In the present study, we investigated the protein and microRNA content of exosomes isolated from the blood serum and PPM urine of CP/CPPS type IIIb patients and healthy men, analyzed their potential to impact gene expression in monocytes and endothelial cells, and performed *in silico* functional studies to characterize the disease ontology of CP/CPPS and to reveal affected pathways. Our analyses of exosomes obtained from PPM urine samples from men with CP/CPPS and healthy men provide profound insight into the conditional state and microenvironment of the prostate in CP/CPPS.

## 2 Materials and methods

### 2.1 Patient and control samples

Blood serum and PPM urine samples were collected from patients diagnosed with CP/CPPS type IIIb (in total *n* = 25, median age 50.9 years, range 23.9–65.7 years) and from healthy men without any pre‐existing urological conditions (in total *n* = 25, median age 31.4 years, range 23.2–48.3 years) in the period between 2014 and 2017 at the Clinic of Urology, Pediatric Urology and Andrology, Justus‐Liebig‐University Giessen, Germany. All biological samples were stored at −80 °C within 1 h after collection and remained there until used in experiments. All patients and healthy probands were informed about the study and provided written consent. The study was approved by the Ethics Commission of the Medical Faculty of Justus‐Liebig‐University Giessen (Ethical Vote, AZ 55/13). All study methodologies conformed to the standards set by the Declaration of Helsinki.

The Chronic Prostatitis Symptom Index (CPSI), comprising indices of pain (CPSI‐1), urination (CPSI‐2), and quality of life (CPSI‐3), was recorded for all study participants, as well as the International Prostate Symptom Score (IPSS), a questionnaire for identification of BPH, and detailed information on urination (Table 1). Semen parameters, including sperm characteristics (concentration, motility, and vitality), pH, number of peroxidase‐positive cells, and biochemical parameters, such as the concentrations of elastase, α‐glycosidase, fructose, zinc, and interleukin‐8, were also determined for all study participants (Table 1). Semen and urine samples of CP/CPPS patients were routinely analyzed by PCR for infections with *Neisseria gonorrhoeae*, *Chlamydia trachomatis*, *Mycoplasma genitalum*, *Mycoplasma hominis*, *Ureaplasma urealyticum*, and *Trichomonas vaginalis*. Blood parameters, such as C‐reactive protein (CRP), PSA, and testosterone, were available for the patient group (Table 1).

**Table 1:** Clinical data of analyzed CP/CPPS patients and healthy control probands. Sperm motility‐a, ‐b, ‐c, and ‐d (rapidly progressive, slowly progressive, non‐progressive, and immotile, respectively). Min, minimum; Max, maximum; Conc., concentration; mL, milliliter; dL, deciliter.

Clinical parameters	CP/CPPS patients (*n* = 25), median (min–max)	Healthy controls (*n* = 25), median (min–max)	Asymptotic significance^a^
CPSI‐1 (pain)	9.0 (0.0–19)	0.0 (0.0–4)	< 0.001
CPSI‐2 (urination)	2.0 (0.0–9)	1.0 (0.0–3)	0.006
CPSI‐3 (quality of life)	6.0 (2.0–12)	0.0 (0.0–3)	< 0.001
CPSI (total score)	17.0 (0.0–34)	1.0 (0.0–8)	< 0.001
IPSS	7 (0.0–28)	2 (0.0–7)	< 0.001
Semen volume (mL)^b^	2.2 (0.1–6.3)	2.9 (0.5–4.9)	0.389
Semen pH value^b^	7.6 (7.1–10)	8.3 (8.1–8.7)	< 0.001
Total sperm count (10^6^)^b^	28.1 (0.0–921.7)	127.5 (15.3–725.2)	0.003
Sperm conc. (10^6^ mL^−1^)^b^	23.6 (0.2–342.5)	45.4 (15.4–148)	0.168
Sperm motility‐a (%)^b^	12 (0.0–44.0)	42.5 (14.0–72.0)	< 0.001
Sperm motility‐b (%)^b^	23 (0.0–36.0)	21.5 (14.0–27.0)	0.325
Sperm motility‐c (%)^b^	13 (0.0–19.0)	11 (3.0–22.0)	0.658
Sperm motility‐d (%)^b^	47.5 (28.0–100.0)	25 (11.0–53.0)	< 0.001
Sperm vitality (%)^b^	61 (0.0–85.0)	86 (62.0–97.0)	< 0.001
Peroxidase‐positive (10^6^ mL^−1^)^b^	0.1 (0.0–0.8)	0.1 (0.0–1.5)	0.178
Immature sperm (10^6^ mL^−1^)^b^	2 (0.0–46.6)	0.0 (0–2.3)	< 0.001
Fructose (μmol per ejaculate)^b^	13 (0.3–129)	45.1 (9.8–116.6)	< 0.001
α‐Glucosidase (mU per ejaculate)^b^	17.2 (2.8–83.6)	42 (19.6–134.8)	< 0.001
Elastase (ng·mL^−1^)	65.5 (5.0–2000.0)	45.0 (5.0–415.0)	0.161
Zinc (μmol per ejaculate)	4.5 (0.1–21.6)	7.3 (2.2–22.6)	0.021
Interleukin‐8 (pg·mL^−1^)	3694 (9.8–21 941)	1864 (458–20 226)	0.023
CRP (mg·L^−1^)	1.12 (0.5–9.25)	Normal reference ≤ 5 mg·L^−1^	
PSA (ng·mL^−1^)	2.4 (0.38–7.75)	Normal reference ≤ 4 ng·mL^−1^	
Testosterone (ng·dL^−1^)	428 (221–714)	Normal reference > 300 ng·dL^−1^	

^a^ Mann–Whitney *U* test (two‐sided *P*‐value).

^b^ Semen analysis was done according to WHO‐2010 laboratory manual for the examination and processing of human semen.

### 2.2 Isolation of exosomes from blood serum and post‐prostatic‐massage urine

Blood serum (2 × 500 μL) and PPM urine (2 × 2 mL) samples from each study participant were thawed on ice. Urine samples were centrifuged at 2000 ***g*** for 15 min at 4 °C to remove cells and cell debris. Blood serum exosomes were isolated using the Total Exosome Isolation Reagent for serum (Invitrogen, Carlsbad, CA, USA; cat. no. 4478360), and PPM urine exosomes were isolated using the Total Exosome Isolation Reagent for urine (Invitrogen; cat. no. 4484452) according to the manufacturer's protocols. Exosomes were resuspended in 150 μL of ice‐cold Exosome Resuspension Buffer (Invitrogen); in the end, we had two 150 μL samples of blood serum exosomes and two 150 μL samples of PPM urine exosomes for each study participant for RNA and protein isolation. Due to the cell toxicity of the exosome resuspension buffer, exosomes were resuspended in 150 μL of autoclaved ddH_2_O prior to treatment of the THP‐1 human monocyte cell line, human umbilical vein endothelial cells (HUVECs), and human primary endothelial cells (ECs). Isolated exosomes were verified using transmission electron microscopy (TEM). For TEM analysis of PPM urine exosomes, 20 μL (of 150 μL) of ddH_2_O exosome suspension was used. For TEM analysis of blood serum exosomes, 3 μL (of 150 μL) of ddH_2_O exosome suspension was further 1 : 20 diluted with ddH_2_O. Formvar‐coated mesh grids were placed first on ddH_2_O exosome suspensions for 30 s, then on 1% ammonium heptamolybdate solution for 1 min, and examined by TEM (Zeiss EM902, Oberkochen, Germany) with 85 000× and 140 000× magnification (Fig. S1). Digital images were captured with a slow‐scan 2K CCD camera (TRS, Tröndle, Moorenweis, Germany).

### 2.3 RNA and protein isolation from exosomes

Exosome RNA and protein were isolated from the exosome samples obtained as outlined above using the Total Exosome RNA & Protein Isolation Kit (Invitrogen; cat. no. 4478545) according to the manufacturer's protocol. Exosome RNA was eluted in 50 μL of preheated elution solution and the RNA concentration was measured using a NanoDrop™ 1000 Spectrophotometer (Thermo Fisher Scientific, Waltham, MA, USA). Exosome protein was measured directly in exosome resuspension buffer using the Pierce® BCA Protein Assay Kit (Thermo Fisher Scientific; cat. no. 23225) and Labsystems Multiskan Rc Plate Reader (Thermo Fisher Scientific) and diluted to a working solution of 2 μg·μL^−1^.

### 2.4 Quantitative western blot analysis of exosomal protein

Protein samples isolated from the blood serum exosomes of CP/CPPS patients (*n* = 20 of 25 in total) and healthy men (*n* = 20 of 25 in total) were analyzed for the presence of four different exosome‐specific markers: CD63, CD9, CD81, and HSP70 (heat shock protein 70). CD63, CD9, and CD81 are cell surface proteins of the transmembrane superfamily 4 (tetraspanin family). The protein samples from blood serum exosomes were also analyzed for select factors known to be upregulated in PCa: PSA, fatty acid synthase, and survivin. Protein samples isolated from PPM urine exosomes possessed very low concentrations and were insufficient for western blot analyses.

Briefly, 40 μg of exosome protein was mixed with Laemmli buffer containing 10% β‐mercaptoethanol, incubated at 95 °C for 5 min, and separated by SDS/PAGE (4% stacking gel and 10% separating gel). As a positive control, 40 μg of Human Exosome Lysate Positive Protein Control (System Bioscience, Palo Alto, CA, USA; cat. no. EXOAB‐POS‐1) was loaded on each gel. The proteins were transferred to a polyvinylidene difluoride membrane using the Bio‐Rad Trans‐Blot SD Semi‐Dry Transfer Cell (Bio‐Rad, Feldkirchen, Germany; 30 min at 0.15 amps and 25 V). The amount of total protein on the membranes was observed using the Revert™ 700 Total Protein Stain Kit (LI‐COR Biosciences, Bad Homburg, Germany; cat. no. 926‐11015) according to the manufacturer's protocol. Images of the total protein were taken at 700 nm using the Odyssey® Fc Imaging System (LI‐COR Biosciences). Afterward, the stain was removed using Revert™ Reversal Solution (LI‐COR Biosciences; cat. no. 926‐11013) for 5 min. Subsequently, the membranes were blocked for 60 min in Odyssey® Blocking Buffer (LI‐COR Biosciences; cat. no. 927‐50000), mixed in a 1 : 1 ratio with PBS, and incubated with the primary antibody overnight at 4 °C (Table S1). The membranes were washed four times with PBS containing 0.1% Tween‐20 and incubated with secondary antibodies (Table S1). Both primary and secondary antibodies were diluted in Odyssey® Blocking Buffer containing 0.1% Tween‐20. After four washes with PBS (0.1% Tween‐20), fluorescence signals were detected using the Odyssey® Fc Imaging System, and specific proteins were quantified in proportion to the total amount of protein (Fig. S1) using image studio lite version 5.2 (LI‐COR Biosciences). The Mann–Whitney *U* test was used to compare the relative protein levels in two groups, and *P*‐values < 0.05 were considered significant.

### 2.5 RT‐qPCR analysis of exosomal microRNA

Samples of RNA isolated from blood serum exosomes and PPM urine exosomes from CP/CPPS patients (*n* = 20 of 25 in total) and healthy men (*n* = 20 of 25 in total) were analyzed for select microRNAs known to be upregulated in PCa: hsa‐miR‐141, hsa‐miR‐375, hsa‐miR‐501, and hsa‐miR‐532. Reverse transcription (RT) of 100 ng of RNA into cDNA was achieved using the MystiCq® microRNA cDNA Synthesis Mix Kit (Sigma‐Aldrich, Taufkirchen, Germany; cat. no. MIRRT‐100RXN) according to the manufacturer's protocol. Quantitative PCR (qPCR) was performed using 20 ng cDNA per reaction, commercial primer sets for targeted microRNAs (Table S2), and MystiCq® microRNA® SYBR® Green qPCR ReadyMix™ (Sigma‐Aldrich; cat. no. MIRRM00‐100RXN) on the CFX96 Touch™ RealTime PCR Detection System (Bio‐Rad). *SNORD44* (small nucleolar RNA, C/D Box 44) was utilized as a reference gene in the quantification of exosome microRNAs and was amplified using the Human Positive Control Primer (Sigma‐Aldrich; part of MIRRT‐100RXN). Quantitative PCR was performed in duplicate. Relative expression levels of microRNAs were calculated by the $2^{-\Delta \Delta C_{T}}$ method. The Mann–Whitney *U* test was used to compare the relative microRNA levels in two groups, and *P*‐values < 0.05 were considered significant.

### 2.6 miScript® miRNA PCR Array “Human Prostate Cancer” analysis in urine exosomes

RNA samples isolated from the PPM urine exosomes of CP/CPPS patients (*n* = 3; ages: 52, 24, and 47 years) and healthy men (*n* = 3; ages: 28, 31, and 37 years) exhibited a sufficiently high amount of RNA and could be analyzed using the miScript miRNA PCR Array “Human Prostate Cancer” (Qiagen, Hilden, Germany; cat. no. MIHS‐112Z) (Table S3). This array comprises 84 human microRNAs known to be crucially involved in PCa development and progression. Briefly, RNA samples (125 ng each) were reverse transcribed using the miScript II RT Kit (Qiagen; cat. no. 218160) and applied to the miScript® miRNA PCR Array. PCR arrays were carried out in duplicate for each RNA sample. The determination of whether a microRNA is expressed was set by the lower limit of detection (set *C*_T_ cut‐off < 35; recommended by the manufacturer). Quality checks were also performed, with all samples passing PCR array reproducibility and RT efficiency. Normalization was achieved using the global *C*_T_ mean of expressed microRNAs. This method automatically calculates a global *C*_T_ mean for the microRNA targets that are commonly expressed in all samples being analyzed. Student's *t*‐test of the replicate $2^{-\Delta C_{T}}$ values was used for the calculation of *P*‐values, and *P* < 0.05 was considered significant. Relative microRNA expression levels were calculated according to the manufacturer's (Qiagen) suggestion using the SA Biosciences PCR Array Data Analysis Web portal supplied by SABiosciences (Frederick, MD, USA).

### 2.7 THP‐1 cell culture and polarization

The THP‐1 human monocyte cell line (ATCC, Manassas, VA, USA; cat. no. TIB‐202) was cultured in Roswell Park Memorial Institute (RPMI) 1640 medium (Thermo Fisher Scientific; cat. no. 21875034) supplemented with 10% fetal calf serum (FCS) and 1% penicillin/streptomycin at 37 °C in a 5% CO_2_ atmosphere. Differentiation of THP‐1 macrophages into the resting (M0) state was induced by treatment of the cells with 20 ng·mL^−1^ phorbol‐12‐myristat‐13‐acetate (PMA) for 48 h. After washing the adherent M0 macrophages with PBS, the cells were incubated with FCS‐free RPMI 1640 medium for 24 h at 37 °C in a 5% CO_2_ atmosphere_._ Polarization of THP‐1 (M0) cells into M1 macrophages was induced by incubating the cells with 40 ng·mL^−1^ of interferon‐gamma (IFN‐γ) and 20 pg·mL^−1^ lipopolysaccharide (LPS), and into M2 macrophages by incubation of cells with 40 ng·mL^−1^ interleukin (IL)‐4 and 40 ng·mL^−1^ IL‐13. Polarization was performed for 24 and 48 h. After harvesting the cells, the RNA was isolated using the peqGOLD TriFast™ reagent (VWR, Darmstadt, Germany; cat. no. 30‐2010), reverse transcribed into cDNA using the LunaScript RT SuperMix Kit (New England Biolabs, Frankfurt/Main, Germany; cat. no. E3010S), and polarization checked by quantitative expression analysis of M1 and M2 marker genes (tumor necrosis factor alpha [*TNFα*] and CC‐chemokine ligand 22 [*CCL22*], respectively) using appropriate primers (Table S4) and the Rotor‐Gene SYBR Green PCR Kit (Qiagen; cat. no. 204074). The mRNA expression of glyceraldehyde 3‐phosphate dehydrogenase (*GAPDH*) was used as a reference. The qPCR was performed in duplicate. The Student's *t*‐test was used to compare the expression of *TNFα* in M0 and M1 macrophages and *CCL22* in M0 and M2 macrophages, and *P*‐values < 0.05 were considered significant.

### 2.8 Treatment of THP‐1 cells with blood serum and PPM urine exosomes

Exosomes isolated from the blood serum and PPM urine of CP/CPPS patients and healthy men were added to THP‐1 cells in the M0 state in order to investigate the capacity of exosomes to enter the cells and regulate their gene expression and polarization. Blood serum exosomes from four CP/CPPS patients (ages: 47, 47, 65, and 63 years) and four healthy men (ages: 24, 28, 29, and 31 years) were available for this study. PPM urine exosomes from six CP/CPPS patients (ages: 62, 47, 51, 52, 64, and 24 years) and four healthy men (ages: 29, 32, 30, and 37 years) were available for this study. Briefly, to induce the M0 state, 1 × 10^6^ THP‐1 cells were seeded in six‐well plates in RPMI 1640 medium supplemented with 10% FCS, 1% penicillin/streptomycin, and 20 ng·mL^−1^ PMA and incubated for 48 h at 37 °C in a 5% CO_2_ atmosphere. Subsequently, the PMA‐containing medium was discarded, the cells were washed with PBS, and then incubated with FCS‐free RPMI 1640 medium (recovery period 24 h). Exosome samples (25 μL of exosome suspension in autoclaved ddH_2_O) were mixed with 1.975 mL FCS‐free RPMI 1640 medium and added to THP‐1 cells. We tested treatment times of 24 and 48 h. Untreated and exosome‐treated THP‐1 cells were analyzed by immunofluorescent staining (IF) for exosome‐specific marker CD81, RT‐qPCR for expression of M1 marker *TNFα* and M2 marker *CCL22*, and by RT^2^ Profiler PCR Arrays (Table S3; the detailed description is in the chapter “RT‐qPCR and RT^2^ Profiler PCR Array analysis of THP‐1 cells treated with exosomes”).

### 2.9 Immunocytochemical analysis of CD81 in THP‐1 cells treated with exosomes

The exosome‐specific marker CD81 was analyzed by immunocytochemistry (ICC) in THP‐1 cells treated with blood serum and PPM urine exosomes. After 24 and 48 h of treatment, THP‐1 cells were removed from cell plates using TrypLE Express reagent (Thermo Fisher Scientific; cat. no. 12604013), washed with PBS, and resuspended in 400 μL of PBS. A 100 μL aliquot of the cell suspension was used for IF, and the remaining part (300 μL) was used for RNA isolation and analysis. For IF, the cell suspension was added onto a slide in a circle drawn by a PAP pen as a hydrophobic barrier. The slides were then air dried at room temperature for 1 h. Acetone (100%) was added into a glass tank on ice for pre‐cooling. The dried‐out slides were put into the pre‐cooled acetone and fixed for 20 min. The acetone was removed by washing the slides with PBS for 5 min three times. After blocking the slides in 5% bovine serum albumin (BSA) for 30 min, the first antibody (anti‐CD81; Abcam, Cambridge, UK; cat. no. ab59477) (Table S1) diluted in 5% BSA was added and the slides incubated overnight at 4 °C in a humidified chamber. After washing off the first antibody using PBS (three times for 5 min), the secondary antibody and Hoechst 33342 nucleic acid stain were added (Table S1) and the slides were incubated for 1 h in a humidified dark chamber at room temperature. Slides were washed with PBS for 5 min in the dark three times. The slides were mounted with Faramount Aqueous Mounting Medium (Agilent, Oberhaching, Germany; cat. no. S302580) and a coverslip added. Fluorescence was observed under an Axioscop2 (Zeiss) and visualized using axiovision rel (version 4.7; Zeiss).

The specificity of the CD81 antibody (Abcam; cat. no. ab59477) was validated by immunohistochemistry (IHC) using synovial tissue from osteoarthritis patients (Ethics Commission of the Medical Faculty of Justus‐Liebig‐University Giessen; ethical vote, AZ 66/08). To this end, 5‐μm cryosections were fixed for 10 min in acetone, rinsed with PBS, and blocked with 5% BSA for 60 min. The first antibody, isotype control IgG2a (BD Biosciences, Franklin Lakes, NJ, USA; cat. no. 550339) (Table S1) or PBS control, was added overnight. After 5 min of washing in PBS, the second antibody, goat anti‐mouse IgG Alexa Fluor 488 (Thermo Fisher Scientific; cat. no. A‐11001) (Table S1), was added for 60 min, followed by 4′,6‐diamidino‐2‐phenylindole (DAPI) staining.

### 2.10 RT‐qPCR and RT^2^ Profiler PCR Array analysis of THP‐1 cells treated with exosomes

RNA was isolated from THP‐1 cells treated with blood serum exosomes (*n* = 4 healthy and *n* = 4 CP/CPPS were available for this study) and PPM urine exosomes (*n* = 4 healthy and *n* = 6 CP/CPPS were available for this study) for 24 and 48 h using the peqGOLD TriFast™ reagent (VWR; cat. no. 30‐2010). For RT‐qPCR, 100 ng of RNA was reverse transcribed into cDNA using the LunaScript RT SuperMix Kit (New England Biolabs; cat. no. E3010S) and analyzed for expression of *TNFα* (M1 marker) and *CCL22* (M2 marker) using the Rotor‐Gene SYBR® Green Mix (Qiagen) and appropriate primer sets (Table S4) on the CFX96 Touch™ RealTime PCR Detection System (Bio‐Rad). The housekeeping gene *GAPDH* was used as a reference gene in calculating the relative gene expression values with the $2^{-\Delta \Delta C_{T}}$ method. Expression of *TNFα* and *CCL22* in untreated THP‐1 (M0) cells was considered to be 1, and the fold change in *TNFα* and *CCL22* expression in exosome‐treated THP‐1 cells was determined. PCR was performed in duplicate. Fisher's exact test was used to compare the relative expression of *TNFα* and *CCL22* in THP‐1 cells treated for 24 h with serum and urine exosomes (healthy men versus CP/CPPS patients) and 48 h (healthy men versus CP/CPPS patients). *P*‐values < 0.05 were considered significant.

For the RT^2^ Profiler PCR Array, 1 μg RNA samples from untreated THP‐1 (M0) cells and THP‐1 cells treated for 24 h with PPM urine exosomes from CP/CPPS (*n* = 3) and healthy men (*n* = 2) were reverse transcribed into cDNA using the RT^2^ First Strand Kit (Qiagen; cat. no. 330404) and applied to two RT^2^ Profiler PCR Arrays: “Human Cancer Inflammation and Immunity Crosstalk” (Qiagen; cat. no. PAHS‐181Z) and “Human Transcription Factors” (Qiagen; cat. no. PAHS‐075Z) (Table S3). Each array allows simultaneous analysis of 84 functionally selected genes. The relative gene expression levels were calculated according to the manufacturer's suggestion using the SA Biosciences PCR Array Data Analysis Web portal (Qiagen). Five housekeeping genes (*ACTB*, *B2M*, *GAPDH*, *HPRT1*, and *RPLP0*) were included in each array. The genes *B2M*, *GAPDH*, and *ACTB* showed the most stable results among all samples and were chosen as reference genes for the normalization of the values generated by RT^2^ Profiler. The expression of genes in untreated THP‐1 cells was considered a control reference, and > 2‐fold regulation of genes in exosome‐treated THP‐1 cells was considered significant. Quality checks, including the reverse transcription efficiency and PCR array reproducibility, were included in the array results report, and all samples passed that check.

### 2.11 Treatment of HUVECs and primary endothelial cells with blood exosomes

Human umbilical vein endothelial cells (Promocell, Heidelberg, Germany; cat. no. C‐12200) were cultured in Endothelial Growth Medium 2 (ECM2; Promocell; cat. no. C‐22011) on six‐well plates at 37 °C in a 5% CO_2_ atmosphere. Primary ECs were isolated from varicose veins as previously published in Zimmermann‐Geller et al., 2019 [33] (Ethics Commission of the Medical Faculty of the Justus‐Liebig‐University Giessen; ethical vote, AZ 142/08). After resection, vessels were washed and filled with collagenase H (1 mg·mL^−1^; Roche, Basel, Switzerland) for 1 h at 37 °C. Detached cells were seeded onto rat tail collagen‐coated plates. Purity was confirmed by immunocytochemistry against CD31. ECs were cultured to maximal passage 3 in ECM2 (Promocell; cat. no. C‐22011). HUVECs and primary ECs were stimulated with 5 ng·mL^−1^ TNFα (Peprotech, Hamburg, Germany; cat. no. AF‐300‐01A‐100 μg). In parallel, the same cells were treated with blood serum exosomes (20 μL exosome suspension per well) from CP/CPPS patients (*n* = 5 were available for this study; ages: 32, 46, 48, 26, and 43 years) and healthy men (*n* = 5 were available for this study; ages: 20, 24, 28, 23, and 25) for 24 and 48 h.

The exosome‐specific marker CD81 was analyzed in untreated, TNFα‐stimulated, and exosome‐treated HUVECs and primary endothelial cells by ICC. The cells were seeded on chamber slides and washed with PBS, and the same procedure was followed as described above for IHC experiments on CD81 in synovial tissues.

Endothelial cell activation was analyzed in untreated, TNFα‐stimulated, and exosome‐treated HUVECs and primary endothelial cells by RT‐qPCR based on the expression of marker genes for endothelial cell activation (*ICAM‐1*, *VCAM‐1*, *E‐selectin*, and *P‐selectin*; Table S4). RNA was isolated using the RNeasy Mini Kit (Qiagen; cat. no. 74105), reverse‐transcribed into cDNA using AMV reverse transcriptase (Promega, Fitchburg, WI, USA; cat. no M5101) and random‐hexamer primers, and amplified using PowerUp™ SYBR® Green Mastermix (Thermo Fisher Scientific; cat. no. A25742) on the QuantStudio™ 5 Real‐Time PCR System (Thermo Fisher Scientific). The expression of 18S rRNA was used as a reference control in calculating the relative gene expression values with the $2^{-\Delta \Delta C_{T}}$ method. In addition, endothelial cell activation was analyzed by enzyme‐linked immunosorbent assay (ELISA) based on the expression of marker proteins of endothelial cell activation (IL‐6, activin A, and monocyte chemoattractant protein 1 [MCP‐1]) using the cell culture supernatants (Table S5). Fisher's exact test was used to compare the relative expression of the mentioned genes and proteins in HUVECs and primary endothelial cells treated for 24 h with serum exosomes (healthy men versus CP/CPPS patients) and for 48 h (healthy men versus CP/CPPS patients). *P*‐values < 0.05 were considered significant.

### 2.12 Data sources and statistical analysis

The Cancer Genome Atlas (TCGA) database (tcgaportal.org), particularly the genome‐wide RNA‐sequencing data for 337 human PCa (adenocarcinoma) and 35 normal prostate tissue (NOR) samples, was used to analyze the following: the expression of microRNAs significantly upregulated and downregulated in PPM urine exosomes from CP/CPPS patients in PCa versus NOR; and the expression of putative target genes of microRNAs, which were significantly upregulated in PPM urine exosomes from CP/CPPS patients based on *in silico* analyses (see spidermir below) and RT^2^ Profiler results generated on THP1 cells before and after treatment with urine exosomes, in PCa versus NOR; the correlation between microRNA expression and age; and the mutual correlation between 14 microRNAs showing at least twofold upregulation in PPM exosomes from CP/CPPS patients. The Mann–Whitney *U* test was used to compare the relative expression of microRNAs and their putative target genes in fragments per kilobase per million mapped reads (FPKM) in PCa and NOR. *P*‐values < 0.05 were considered significant. The Spearman correlation method (Spearman's Rho; two‐sided *P*‐value) was used for correlation analyses, and Spearman's Rho > 0.3 was considered significant.

Functional *in silico* studies were performed on microRNAs and the putative target genes of nine microRNAs were found to be significantly upregulated in the PPM urine from CP/CPPS patients. Only genes that were a common target of ≥ 4 upregulated microRNAs were considered (*n* = 825 common target genes). spidermir, an r/bioconductor package for integrative network analysis of microRNA data [34], and dose, an r/bioconductor package for Disease Ontology Semantic and Enrichment (DOSE) analysis [35], were used for the identification and analysis of putative target genes and visualization of networks. spidermir facilitates network open‐access data retrieval from GeneMania data, prepares the data using the appropriate gene nomenclature, and integrates microRNA data in a specific network [34]. Gene Ontology (GO) analysis was carried out using the Database for Annotation, Visualization, and Integrated Discovery (DAVID) [36, 37]. Gene set enrichment analysis (GSEA) was performed using the Molecular Signatures Database (MSigDB) [38]. Pathway analysis was carried out using the Kyoto Encyclopedia of Genes and Genomes (KEGG) [39].

## 3 Results

### 3.1 Characterization of CP/CPPS patients

The CP/CPPS type IIIb cohort analyzed in this study is a typical cross‐section of patients suffering from this disease. Compared to healthy men without any urological conditions, CP/CPPS patients usually complain of strong pelvic pain (*P* < 0.001), problems with urination (*P* < 0.01), and severe impairment of quality of life (*P* < 0.001; Mann–Whitney *U* test, two‐sided *P*‐values; Table 1). With a median IPSS value of 7 (range 0.0–28), CP/CPPS patients have mild symptoms with regard to the risk of BPH (Table 1). Furthermore, CP/CPPS patients present with signs of impaired semen quality, including a reduction in the total sperm count (*P* < 0.01), a decrease in progressive sperm motility (*P* < 0.001) and sperm vitality (*P* < 0.001), and decreased semen pH (*P* < 0.001; Mann–Whitney *U* test, two‐sided *P*‐values; Table 1). Biochemical semen parameters, such as the concentration of fructose (*P* < 0.001), α‐glucosidase (*P* < 0.001), zinc (*P* < 0.03), and IL‐8 (*P* < 0.03), are also significantly impaired (Mann–Whitney *U* test, two‐sided *P*‐values; Table 1). However, most CP/CPPS patients exhibit clinically unremarkable concentrations of CRP (median 1.12 mg·L^−1^, range 0.5–9.25), PSA (median 2.4 ng·mL^−1^, range 0.38–7.75), and testosterone (median 428 ng·dL^−1^, range 221–714; Table 1). None of the CP/CPPS patients exhibited bacteriospermia (< 1000 colony‐forming units per milliliter), and all were negative for sexually transmitted diseases by PCR.

### 3.2 Exosome isolation from liquid biopsies and verification using TEM and marker proteins

Exosomes were examined and verified using TEM based on their expected size of circa 30–150 nm (Fig. S1). Exosomal protein samples isolated from 500 μL of blood serum had a mean concentration of 7.8 μg·μL^−1^ (in total 1155 μg), and the yield of exosomal protein isolated from 2 mL of PPM urine was much lower (mean concentration 0.3 μg·μL^−1^, in total 45 μg). Our pretests showed that at least 40 μg of exosomal protein was needed in each of the seven planned quantitative western blot (qWB) to achieve reliable results; therefore, exosomal protein samples isolated from PPM urine were not suitable for qWB.

**Fig. 1:**
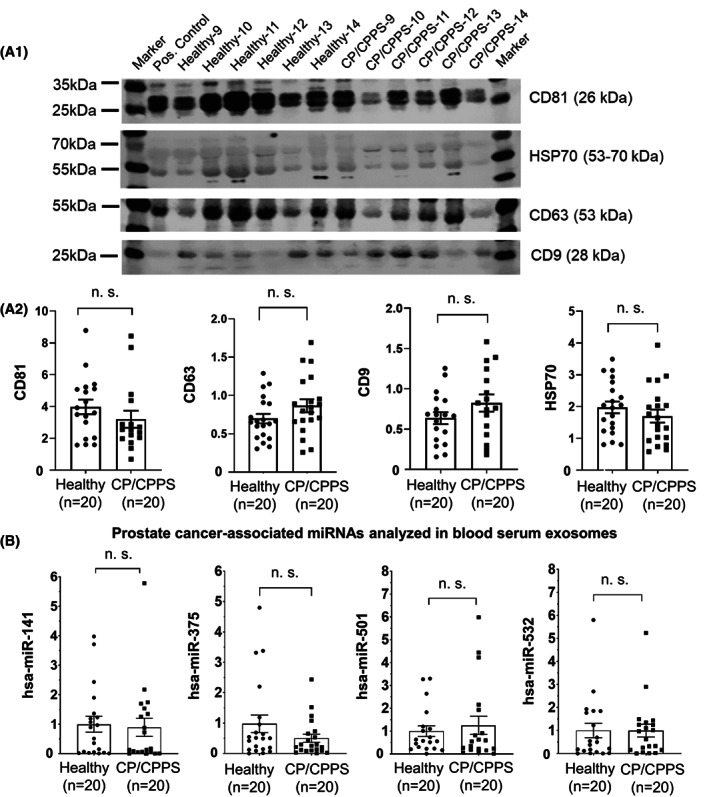
Analysis of exosome‐specific marker proteins and PCa‐associated microRNAs in blood serum exosomes. (A.1) Protein extracts of serum exosomes from healthy men (*n* = 20) and patients with CP/CPPS (*n* = 20) were analyzed by qWB for exosome‐specific marker proteins CD81, HSP70, CD63, and CD9. The Human Exosome Lysate Positive Protein control (System Biosciences) (pos. control) was used to validate the antibodies (exemplary blots presenting six healthy men and six CP/CPPS patients are shown). (A.2) Relative protein levels of CD81, CD63, CD9, and HSP70 in serum exosomes from healthy controls (*n* = 20) and CP/CPPS patients (*n* = 20) were quantified based on total protein amount and compared using the Mann–Whitney *U* test (*P*‐values > 0.05 were considered not significant, n.s.). Data are presented as each value, the mean, and the standard error of the mean. (B) Selected microRNAs known to be elevated in PCa (hsa‐miR‐141, hsa‐miR‐375, hsa‐miR‐501, and hsa‐miR‐532) were analyzed by RT‐qPCR in serum exosomes from healthy men (*n* = 20) and CP/CPPS patients (*n* = 20). The small nucleolar RNA *SNORD44* (amplified using the Human Positive Control Primer; Sigma‐Aldrich) was used as a reference control. The Mann–Whitney *U* test was used, and *P*‐values > 0.05 were considered not significant (n.s.). Data are presented as each value, the mean, and the standard error of the mean.

Exosomal protein from the blood serum of CP/CPPS patients (*n* = 20) and healthy men (*n* = 20) was analyzed by qWB with regard to exosome‐specific tetraspanins CD81, CD63, and CD9 and exosome‐specific heat shock protein HSP70. All exosome‐specific proteins were detectable in patient and control samples (Fig. 1A.1,A.2; Fig. S2A–C), and quantitative analyses showed no differences in the protein levels of CD81, CD63, CD9, and HSP70 between the patient and control groups (Mann–Whitney *U* test; Fig. 1A.2). Furthermore, we analyzed three proteins (i.e., PSA, FAS, and survivin) known to be upregulated in PCa. Faint signals were detected for PSA, whereas FAS and survivin signals were completely absent (Fig. S3A.1,B,C). The qWB analyses showed a moderate increase in PSA in the blood serum exosomes of CP/CPPS patients compared to healthy men (Mann–Whitney *U* test, *P* = 0.0157; Fig. S3A.2).

### 3.3 Upregulation of prostate cancer‐associated microRNAs in CP/CPPS

Exosomal RNA samples isolated from 500 μL of blood serum exhibited a mean concentration of 41 ng·μL^−1^ (50 μL total volume), and those isolated from 2 mL PPM urine exhibited a mean concentration of 23 ng·μL^−1^ (50 μL total volume). All exosomal RNA samples had high purity (mean OD260/280 ratio 1.9).

**Fig. 2:**
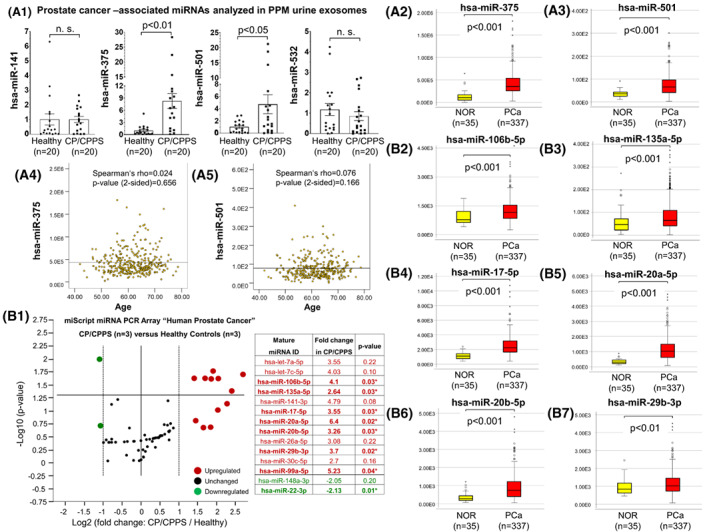
Analysis of PCa‐associated microRNAs in post‐prostatic‐massage (PPM) urine exosomes. (A.1) Selected microRNAs known to be elevated in PCa (hsa‐miR‐141, hsa‐miR‐375, hsa‐miR‐501, and hsa‐miR‐532) were analyzed in PPM urine exosomes from healthy men (controls, *n* = 20) and CP/CPPS patients (*n* = 20) and differences evaluated by the Mann–Whitney *U* test (*P*‐values > 0.05 were considered not significant, n.s.). Data are presented as each value, the mean, and the standard error of the mean. (A.2, A.3) MicroRNAs found to be significantly increased in PPM urine exosomes from CP/CPPS patients (has‐miR‐375 and hsa‐miR‐501) were analyzed in PCa (*n* = 337) and normal prostate tissues (*n* = 35) using the genome‐wide RNA‐sequencing data recorded in TCGA (Mann–Whitney *U* test, *P*‐values are given). Median values are given with a 95% confidence interval and lower and upper limits. (A.4, A.5) To check whether the significantly increased expression of microRNAs in the PPM urine exosomes from CP/CPPS patients (hsa‐miR‐375 and hsa‐miR‐501) correlated with age, Spearman's correlation analysis was performed using the TCGA dataset (PCa, *n* = 337). Spearman's rank correlation coefficient Rho, two‐sided *P*‐values, and linear regression line are given. (B.1) RNA samples isolated from the PPM urine exosomes from healthy men (controls, *n* = 3) and CP/CPPS patients (*n* = 3) were analyzed by miScript® miRNA PCR Array “Human Prostate Cancer” (Qiagen). The Volcano plot and list of microRNAs exhibiting a ≥ 2‐fold change in the PPM urine exosomes of CP/CPPS patients are given (red: upregulated microRNAs; green: downregulated microRNAs). Significantly upregulated and downregulated microRNAs are highlighted in bold (Student's *t*‐test, *P < 0.05) (horizontal bar: significance threshold). (B.2–B.7) Seven microRNAs found to be significantly upregulated in the PPM urine exosomes from CP/CPPS patients (shown in B.1) were analyzed in PCa (*n* = 337) and normal prostate tissues (*n* = 35) using the genome‐wide RNA‐sequencing data recorded in TCGA. Six of seven analyzed microRNAs were also significantly upregulated in PCa (Mann–Whitney *U* test, *P* < 0.05 was considered significant). Median values are given with a 95% confidence interval and lower and upper limits.

Based on the literature, we selected four microRNAs (hsa‐miR‐141, hsa‐miR‐375, hsa‐miR‐501, and hsa‐miR‐532) that are presumably upregulated in prostate carcinogenesis. After validation of RT‐qPCR primer sets (Fig. S4), we analyzed RNA samples isolated from blood serum and PPM urine exosomes with regard to these microRNAs. We found no difference between CP/CPPS patients and healthy men for all four microRNAs in serum exosomes (Mann–Whitney *U* test; Fig. 1B). In contrast, in PPM urine exosomes, we found a significant increase in hsa‐miR‐375 (*P* < 0.01) and hsa‐miR‐501 (*P* < 0.05) in CP/CPPS patients, whereas hsa‐miR‐141 and hsa‐miR‐532 exhibited similar levels in patients and controls (*P* > 0.05, Mann–Whitney *U* test; Fig. 2A.1). Next, using TCGA datasets, we analyzed the expression of hsa‐miR‐375, hsa‐miR‐501, hsa‐miR‐141, and hsa‐miR‐532 in a representative cohort of PCa (*n* = 337) and NOR (*n* = 35) and confirmed their significant upregulation in PCa specimens (Mann–Whitney *U* test, *P* < 0.001 for all four microRNAs; Fig. 2A.2,A.3; Fig. S5A,B). As our CP/CPPS patient group was older than the healthy control group, we checked whether the expression of hsa‐miR‐375 and hsa‐miR‐501 correlates with age. We used the TCGA dataset of 337 PCa specimens and performed a Spearman correlation analysis. Neither hsa‐miR‐375 nor hsa‐miR‐501 correlated with age (Spearman's Rho < 0.1 and two‐sided *P*‐value > 0.1 for both microRNAs; Fig. 2A.4,A.5; Table S6).

To gain a comprehensive insight into PPM urine exosomes with regard to the presence and quantities of PCa‐associated microRNAs, we analyzed the RNA from PPM urine exosomes from CP/CPPS patients and healthy controls using the miScript microRNA PCR Array “Human Prostate Cancer” comprising 84 microRNAs crucially involved in prostate carcinogenesis. Forty‐one of 84 analyzed microRNAs were determinable and quantifiable, and the rest were either absent or detectable in very low amounts (Table S7). We detected 12 PCa‐associated microRNAs exhibiting > 2‐fold upregulation (from 2.7 to 6.4) in all three analyzed CP/CPPS patients compared to healthy men (Fig. 2B.1). The upregulation of 7 of 12 microRNAs (hsa‐miR‐106b, hsa‐miR‐135a, hsa‐miR‐17, hsa‐miR‐20a, hsa‐miR‐20b, hsa‐miR‐29b, and hsa‐miR‐99a) was significant (Student's *t*‐test; Fig. 2B.1). Two microRNAs, hsa‐miR‐148a and hsa‐miR‐22, showed > 2‐fold downregulation in the PPM urine exosomes of CP/CPPS patients, whereas the downregulation of hsa‐miR‐22 was significant (Student's *t*‐test, *P* = 0.01; Fig. 2B.1). When comparing each patient to the control group, we found several PCa‐associated microRNAs were individually > 2‐fold upregulated or downregulated in CP/CPPS (Fig. S6A–C). Next, the expression of seven microRNAs found to be significantly upregulated in PPM urine exosomes from CP/CPPS patients (Fig. 2B.1) was analyzed using TCGA datasets for a representative cohort of PCa (*n* = 337) and NOR (*n* = 35). Six of seven microRNAs (hsa‐miR‐106b, hsa‐miR‐135a, hsa‐miR‐17, hsa‐miR‐20a, hsa‐miR‐20b, and hsa‐miR‐29b) were also significantly upregulated in PCa (Mann–Whitney *U* test, *P* < 0.001 for four microRNAs, and *P* < 0.01 for two microRNAs; Fig. 2B.2–B.7). However, hsa‐miR‐99a expression was significantly downregulated in PCa (*P* < 0.01), and hsa‐miR‐22 did not show a difference between PCa and NOR (*P* = 0.498, Mann–Whitney *U* test; Fig. S5C,D).

### 3.4 Prediction of altered pathways in CP/CPPS based on upregulated microRNAs

**Fig. 3:**
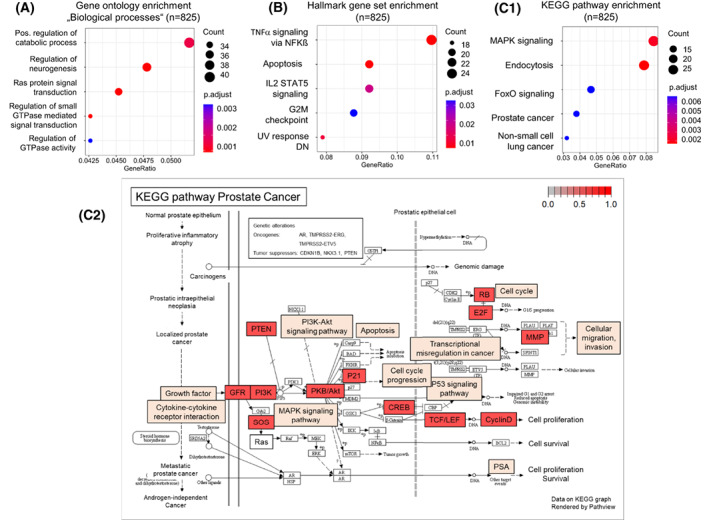
Functional *in silico* analysis of the putative target genes of significantly upregulated microRNAs in CP/CPPS. A total of 825 genes were common putative target genes of at least four significantly upregulated microRNAs in the PPM urine exosomes from CP/CPPS patients (hsa‐miR‐375, hsa‐miR‐501, and seven microRNAs shown in Fig. 2B.1) and analyzed with regard to Gene Ontology enrichment “Biological processes” (A), hallmark gene set enrichment based on the Molecular Signature Database (B), and pathway enrichment based on the Kyoto Encyclopedia of Genes and Genomes (KEGG) database (C.1, C.2) (*P*‐values adjusted by Benjamini–Hochberg method and numbers of counted genes are given). (C.2) Genes enriched in the KEGG pathway PCa (in red) are shown in the context of the whole pathway and individual involved processes, respectively.

To explore the potentially altered pathways in CP/CPPS, we performed *in silico* functional studies on the putative target genes of nine microRNAs found to be significantly upregulated in PPM urine exosomes from CP/CPPS patients (hsa‐miR‐375, hsa‐miR‐501, hsa‐miR‐106b, hsa‐miR‐135a, hsa‐miR‐17, hsa‐miR‐20a, hsa‐miR‐20b, hsa‐miR‐29b, and hsa‐miR‐99a). A total of 1849 putative target genes genome‐wide for the nine upregulated microRNAs in CP/CPPS. Of these genes, 825 were common target genes of at least four upregulated microRNAs and were subjected to *in silico* functional analyses. First, GO enrichment analysis with regard to “Biological processes” showed significant enrichment of the terms “Regulation of neurogenesis” (Benjamini–Hochberg adjusted *P*‐value = 8.87E‐04), “Ras protein signal transduction” (*P* = 8.87E‐04), “Regulation of small GTPase mediated signal transduction” (*P* = 8.87E‐04), and “Positive regulation of cellular catabolic process” (*P* = 1.54E‐03; Fig. 3A; Table S8). Second, Hallmark GSEA showed significant enrichment of the “Hallmark TNFα signaling via NF‐Kβ” (Benjamini–Hochberg adjusted *P*‐value = 1.48E‐03), “Hallmark Apoptosis” (*P* = 1.78E‐03), “Hallmark IL2 STAT5 signaling” (*P* = 1.68E‐02), “Hallmark UV response DN” (*P* = 6.77E‐03), and “Hallmark G2M checkpoint” (*P* = 3.32E‐02; Fig. 3B; Table S9). Third, KEGG pathway enrichment analysis showed significant enrichment of “MAPK signaling pathway” (Benjamini–Hochberg adjusted *P*‐value = 2.11E‐03), “Endocytosis” (*P* = 1.71E‐03), “FoxO signaling pathway” (*P* = 6.89E‐03), “Prostate cancer” (*P* = 6.89E‐03), and “Non‐small cell lung cancer” (*P* = 6.89E‐03; Fig. 3C.1; Table S10). Detailed analysis of the enriched KEGG pathway “Prostate cancer” showed that 13 genes involved in prostate carcinogenesis (*AKT3*, *CCND1*, *CDKN1A*, *CREB5*, *E2F1*, *E2F2*, *MMP3*, *PDGFRA*, *PIK3R1*, *PTEN*, *RB1*, *SOS1*, and *TCF7L1*) may be dysregulated in CP/CPPS due to the upregulation of microRNAs (Fig. 3C.2).

### 3.5 Capacity of urine exosomes in CP/CPPS patients to invade THP‐1 cells and affect their polarization

The intrusion of exosomes into THP‐1 cells was checked on the basis of exosome marker protein CD81. The specificity of the CD81 antibody was positively validated by IHC in CD81‐positive synovial tissue from osteoarthritis patients (Fig. S7). The polarization of THP‐1 cells was analyzed using validated primer sets targeting two representative marker genes, *TNFα* (M1 marker) and *CCL22* (M2 marker; Fig. S8). As a control, we treated THP‐1 cells with IFN‐γ and LPS for M1 polarization, and with IL‐4 and IL‐13 for M2 polarization. A significant increase in *TNFα* was detected in THP‐1 cells after treatment with IFN‐γ and LPS but not after treatment with IL‐4 and IL‐13 (Fig. S9A). Accordingly, a significant increase in *CCL22* expression was detected in THP‐1 cells after treatment with IL‐4 and IL‐13 but not after treatment with IFN‐γ and LPS (Fig. S9B).

**Fig. 4:**
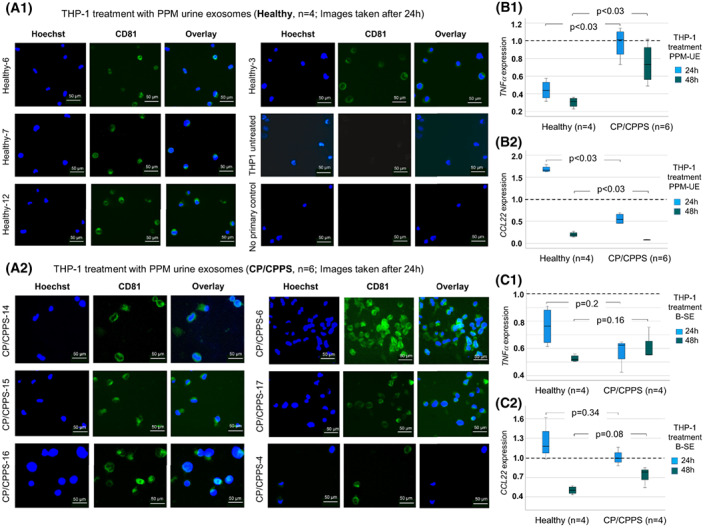
Treatment of the human monocytic cell line THP‐1 with post‐prostatic‐massage urine exosomes. (A.1, A.2) THP‐1 cells in M0 state were incubated with PPM urine exosomes from healthy men (*n* = 4) (A.1) and CP/CPPS patients (*n* = 6) (A.2) for 24 h. Immunofluorescent images were taken of CD81 (exosome‐specific marker, green) and Hoechst (nuclear, blue) staining. Untreated THP‐1 (M0) cells were used as a reference control, and THP‐1 (M0) cells incubated with only secondary antibody (no primary control) were used as a secondary antibody control. The scale bar (50 μm) is shown for each image. (B.1, B.2) THP‐1 cells were incubated with PPM urine exosomes (PPM‐UE) extracted from healthy men (*n* = 4) and CP/CPPS patients (*n* = 6), and the mRNA expression of *TNFα* (a marker of M1 macrophages) (B.1) and *CCL22* (a marker of M2 macrophages) (B.2) was analyzed after 24 and 48 h of treatment. Expression of *TNFα* and *CCL22* in untreated THP‐1 cells (M0 state) was set to 1 (dashed line). Fold changes in *TNFα* and *CCL22* expression in THP‐1 cells treated with PPM urine exosomes extracted from healthy men and CP/CPPS patients were compared (Fisher's exact test, *P* < 0.05 was considered significant). Median values and ranges are given. (C.1, C.2) For comparison to urine exosomes, THP‐1 cells were also incubated with blood serum exosomes (B‐SE) from healthy men (*n* = 4) and CP/CPPS patients (*n* = 4), and the mRNA expression of *TNFα* (C.1) and *CCL22* (C.2) was analyzed after 24 and 48 h of treatment. Expression of *TNFα* and *CCL22* in untreated THP‐1 cells (M0 state) was set to 1 (dashed line). Fold changes in *TNFα* and *CCL22* expression in THP‐1 cells treated with serum exosomes from healthy men and CP/CPPS patients were compared (Fisher's exact test, *P* < 0.05 was considered significant). Median values and ranges are given.

To analyze the potential of exosomes derived from CP/CPPS patients to invade immune cells and affect their gene expression, we incubated THP‐1 cells (M0 state) with PPM urine and blood serum exosomes from CP/CPPS patients and healthy men for 24 and 48 h (Fig. 4; Figs S10 and S11). In untreated THP‐1 cells, the CD81 signal was very weak. After 24 h of incubation, a clear CD81 signal was detected in THP‐1 cells treated with PPM urine exosomes from CP/CPPS patients or healthy men (Fig. 4A.1,A.2). After 48 h, the CD81 signals were still detectable but the intensity slightly diminished (Fig. S10). However, THP‐1 cells treated with blood serum exosomes exhibited markedly lower CD81 signals than those treated with PPM urine exosomes (Fig. S11A,B). Interestingly, PPM urine exosomes from CP/CPPS patients and healthy men had significantly different (opposing) effects on *TNFα* and *CCL22* expression in THP‐1 (Fisher's exact test, *P* < 0.03; Fig. 4B.1,B.2). Although PPM urine exosomes from healthy men were able to suppress *TNFα* and increase *CCL22* in THP‐1 cells after 24 h of incubation, exosomes from CP/CPPS patients did not have altered *TNFα* expression (still similar to M0 state) but decreased *CCL22* expression (Fig. 4B.1,B.2). However, after 48 h, the expression of both genes declined in both groups, but the significant difference was still observable. In contrast, the effect of blood serum exosomes on *TNFα* and *CCL22* expression in THP‐1 cells did not differ significantly between patients and controls (Fisher's exact test, *P* > 0.05; Fig. 4C.1,C.2).

### 3.6 Incapacity of blood exosomes from CP/CPPS patients to activate endothelial cells

**Fig. 5:**
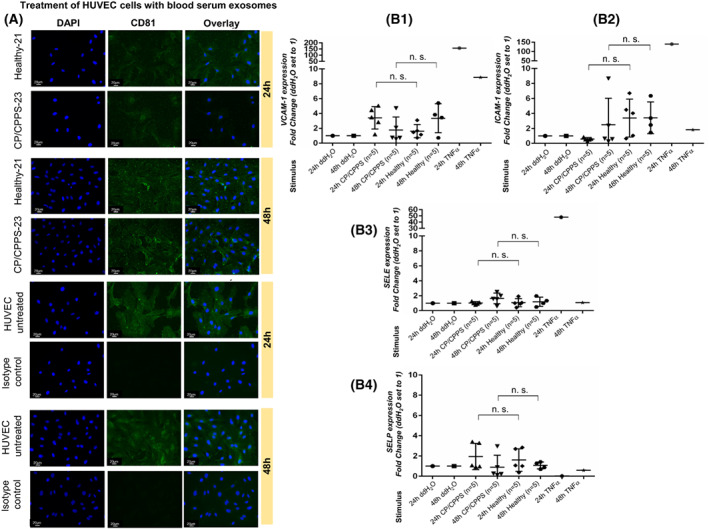
Treatment of HUVECs with blood serum exosomes. (A) HUVECs were incubated with serum exosomes from healthy men (*n* = 5) and CP/CPPS patients (*n* = 5) for 24 h (upper panel) and 48 h (lower panel). Immunofluorescent images were taken of CD81 (exosome‐specific marker, green) and DAPI (nuclear, blue) staining. Exemplary images are shown. Untreated HUVECs were used as a reference control. Isotype control served as the primary antibody control. The scale bar (20 μm) is shown for each image. (B.1–B.4) The mRNA expression of endothelial cell activation markers *VCAM‐1* (B.1), *ICAM‐1* (B.2), *SELE* (B.3), and *SELP* (B.4) was analyzed in untreated HUVECs (24 h ddH_2_O and 48 h ddH_2_O), HUVECs treated with serum exosomes from healthy men (*n* = 5) and CP/CPPS patients (*n* = 5) (24 and 48 h), and HUVECs treated with *TNFα* (24 and 48 h). Fisher's exact test was used to compare the expression of genes in the “Healthy” and “CP/CPPS” groups, and *P* > 0.05 was considered not significant (n.s.). Error bars indicate the standard deviation.

Next, we analyzed whether blood serum exosomes are able to enter endothelial cells and affect their gene expression. We incubated HUVECs and primary endothelial cells with blood serum exosomes from healthy men and CP/CPPS patients and analyzed the expression of representative marker genes and proteins for endothelial cell activation. Our ICC analyses showed that untreated HUVECs possess moderate‐endogenous CD81 expression. Treatment of HUVECs with blood serum exosomes for 24 and 48 h did not markedly change the intensity of the CD81 signals within the cells (Fig. 5A). However, after 48 h of IF, many single green spots were observed in treated HUVECs (Fig. 5A, bottom panel). None of the analyzed marker genes (*VCAM‐1*, *ICAM‐1*, *SELE*, and *SELP*) and proteins (activin A, IL‐6, and MCP‐1) showed a significant change in expression in HUVECs after 24 and 48 h of treatment with blood serum exosomes from patients and controls (Fisher's exact test, *P* > 0.05; Fig. 5B.1–B.4; Fig. S12A–C). The treatment of primary endothelial cells with blood serum exosomes from patients and controls did not show an effect on the expression of markers of endothelial cell activation (Fisher's exact test, *P* > 0.05; Fig. S13).

### 3.7 Altered gene expression in THP‐1 cells after treatment with urine exosomes

**Fig. 6:**
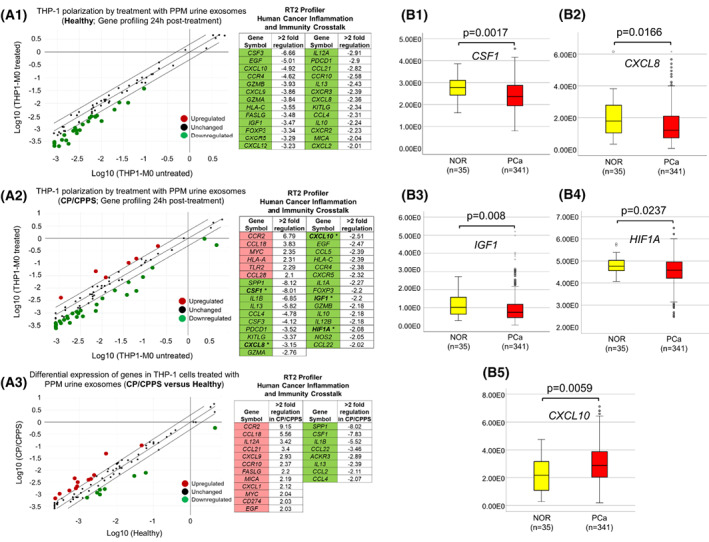
RT^2^ Profiler PCR Array “Human cancer inflammation and immunity crosstalk” analysis in THP‐1 cells. (A.1–A.3) Untreated THP‐1 (M0 state) and THP‐1 cells treated with post‐prostatic‐massage (PPM) urine exosomes from healthy men (*n* = 2) and CP/CPPS patients (*n* = 3) for 24 h were analyzed with regard to the expression of 84 genes crucially involved in human cancer inflammation and immunity crosstalk (Qiagen). Each RNA sample was analyzed in duplicate (i.e., on two arrays). Log10 enrichment of gene expression in treated versus untreated THP‐1 cells was calculated for the healthy group (A.1) and CP/CPPS group (A.2) and depicted in scatter plots. More than twofold regulated genes were considered relevant and were listed in tables next to the plots. Five genes (*CSF1*, *CXCL8*, *CXCL10*, *IGF1*, and *HIF1A*), which were identified as downregulated putative target genes of significantly upregulated microRNAs in PPM urine exosomes from CP/CPPS patients, are marked in bold and asterisk (table in A.2). (A.3) In addition, log_10_ enrichment of gene expression in THP‐1 cells treated with PPM urine exosomes from CP/CPPS patients was calculated in relation to THP‐1 cells treated with exosomes from healthy men. (B.1–B.5) Five genes, which were identified as downregulated putative targets of significantly upregulated microRNAs in PPM urine exosomes extracted from CP/CPPS patients (*CSF1*, *CXCL8*, *HIF1A*, *IGF1*, and *CXCL10*), were analyzed in PCa (*n* = 341) and normal prostate tissue (NOR, *n* = 35) using TCGA dataset. The Mann–Whitney *U* test was used to compare the mRNA expression levels (*y*‐axis: fragments per kilobase million), and *P* < 0.05 was considered significant. Median values are given with a 95% confidence interval and lower and upper limits.

To obtain a broader understanding of the potential adverse effects of CP/CPPS‐associated exosomes on gene expression and assess the risk of CP/CPPS in terms of cancer‐promoting inflammation, we analyzed the RNA of THP‐1 cells treated in the M0 state with PPM urine exosomes from CP/CPPS patients and healthy controls using the RT^2^ Profiler PCR Array “Human cancer inflammation and immunity crosstalk” (Fig. 6A; Table S11). This array allowed simultaneous analysis of the expression of 84 genes involved in cancer inflammation and immunity crosstalk. Compared to the M0 state, THP‐1 cells treated with PPM urine exosomes from healthy men had no upregulated genes and 26 genes that were > 2‐fold downregulated (Fig. 6A.1). In contrast, THP‐1 cells treated with CP/CPPS‐derived PPM urine exosomes had six genes that were > 2‐fold upregulated compared to the M0 state (*CCR2*, *CCL18*, *MYC*, *HLA‐A*, *TLR2*, and *CCL28*; Fig. 6A.2). However, CP/CPPS‐derived PPM urine exosomes were also able to downregulate 25 genes, 15 of which overlapped with downregulated genes in the healthy control group, and 10 were downregulated exclusively in the CP/CPPS group (*SPP1*, *CSF1*, *IL1B*, *CCl4*, *CCL5*, *IL1A*, *IL12B*, *HIF1A*, *NOS2*, and *CCL22*; Fig. 6A.2). A direct comparison of the two groups with regard to their effect on THP‐1 cells revealed 12 overexpressed genes (strongest: *CCR2*, 9.15‐fold; *CCL18*, 5.56‐fold; *IL12A*, 3.42‐fold; and *CCL21*, 3.4‐fold) and eight suppressed genes (strongest: *SPP1*, −8.02‐fold; *CSF1*, −7.83‐fold; *IL1B*, −5.52‐fold; and *CCL22*, −3.46‐fold) in the CP/CPPS group (Fig. 6A.3). Next, we analyzed which of the downregulated inflammatory genes detected in THP‐1 cells after treatment with PPM urine exosomes from CP/CPPS patients (THP‐1‐M0 versus THP‐1‐treated; Fig. 6A.2) represent putative target genes of microRNAs that are significantly upregulated in CP/CPPS (Fig. 2A.1,B.1). Five downregulated inflammatory genes (*CSF1*, *CXCL8*, *CXCL10*, *IGF1*, and *HIF1A*) were identified as putative target genes of at least one of nine microRNAs upregulated in CP/CPPS. Interestingly, analysis of these five genes in PCa and NOR using TCGA datasets showed that *CSF1* (*P* = 0.0017), *CXCL8* (*P* = 0.0166), *IGF1* (*P* = 0.008), and *HIF1A* (*P* = 0.0237) were also significantly downregulated in PCa (Mann–Whitney *U* test; Fig. 6B.1–B.4). *CXCL10* exhibited an opposite trend compared to THP‐1 cells treated with PPM urine exosomes from CP/CPPS patients and was significantly upregulated in PCa (*P* = 0.0059, Mann–Whitney *U* test; Fig. 6B.5).

**Fig. 7:**
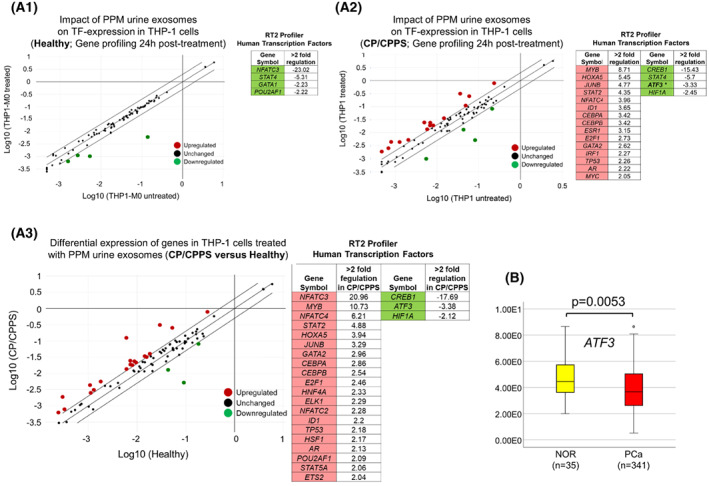
Profiler PCR Array “Human transcription factors” analysis in THP‐1 cells. (A.1–A.3) Untreated THP‐1 (M0 state) and THP‐1 cells treated with post‐prostatic‐massage (PPM) urine exosomes from healthy men (*n* = 2) and CP/CPPS patients (*n* = 3) for 24 h were analyzed with regard to the expression of 84 genes encoding transcription factors (Qiagen). Each RNA sample was analyzed in duplicate (i.e., on two arrays). Log_10_ enrichment of gene expression in treated versus untreated THP‐1 cells was calculated for the healthy group (A.1) and CP/CPPS group (A.2) and depicted in scatter plots. More than twofold regulated genes were considered relevant and are listed in tables next to the plots. One gene (*ATF3*), which was identified as downregulated putative target gene of significantly upregulated microRNAs in PPM urine exosomes extracted from CP/CPPS patients is marked in bold and asterisk (table in A.2). (A.3) In addition, log_10_ enrichment of gene expression in THP‐1 cells treated with PPM urine exosomes from CP/CPPS patients was calculated in relation to THP‐1 cells treated with exosomes from healthy men. (B) One gene identified as downregulated putative target of significantly upregulated microRNAs in PPM urine exosomes from CP/CPPS patients (*ATF3*) was analyzed in PCa (*n* = 341) and normal prostate tissue (NOR, *n* = 35) using TCGA dataset. The Mann–Whitney *U* test was used to compare the mRNA expression levels (*y*‐axis: fragments per kilobase million), and *P* < 0.05 was considered significant. Median values are given with a 95% confidence interval and lower and upper limits.

To analyze whether CP/CPPS‐derived PPM urine exosomes possess the capacity to affect the expression of transcription factors (TFs), we analyzed RNA from THP‐1 cells treated with PPM urine exosomes from CP/CPPS patients and healthy men using the RT^2^ Profiler PCR Array “Human transcription factors” (Fig. 7A; Table S12). This array allowed simultaneous analysis of the expression of 84 genes encoding TFs. Compared to the M0 state, THP‐1 cells treated with PPM urine exosomes from healthy men had zero upregulated genes and four TFs that were > 2‐fold downregulated (Fig. 7A.1). In contrast, THP‐1 cells treated with CP/CPPS‐derived PPM urine exosomes had 15 TFs that were > 2‐fold upregulated (*MYB*, *HOAXA5*, *JUNB*, *STAT2*, *NFATC4*, *ID1*, *CEBPA*, *CEBPB*, *ESR1*, *E2F1*, *GATA2*, *IRF1*, *TP53*, *AR*, and *MYC*) and 4 TFs that were > 2‐fold downregulated (*CREB1*, *STAT4*, *ATF3*, and *HIF1A*) compared to the M0 state (Fig. 7A.2). A direct comparison of exosomes from the two groups with regard to their effect on the expression of genes encoding TFs revealed that THP‐1 cells treated with CP/CPPS‐derived PPM urine exosomes overexpressed 20 genes (strongest overexpression detected for: *NFATC3*, 20.96‐fold; *MYB*, 10.73‐fold; *NFATC4*, 6.21‐fold; *STAT2*, 4.88‐fold; *HOXA5*, 3.94‐fold; and *JUNB*, 3.94‐fold) and underexpressed 4 genes (strongest underexpression detected for: *CREB1*, −17.69‐fold; *ATF3*, −3.38‐fold; Fig. 7A.3). We also analyzed which of the downregulated TF‐encoding genes detected in THP‐1 cells after their treatment with CP/CPPS‐derived PPM urine exosomes (THP‐1‐M0 versus THP‐1 treated; Fig. 7A.2) represent putative target genes of microRNAs significantly upregulated in CP/CPPS (Fig. 2A.1,B.1). Among downregulated TF‐encoding genes, *ATF3* was identified as putative target gene of at least one of nine microRNAs significantly upregulated in PPM urine exosomes from CP/CPPS patients. Analysis of TCGA datasets showed that *ATF3* was also significantly downregulated in PCa (*P* = 0.0053, Mann–Whitney *U* test; Fig. 7B).

### 3.8 Disease ontology of CP/CPPS

**Fig. 8:**
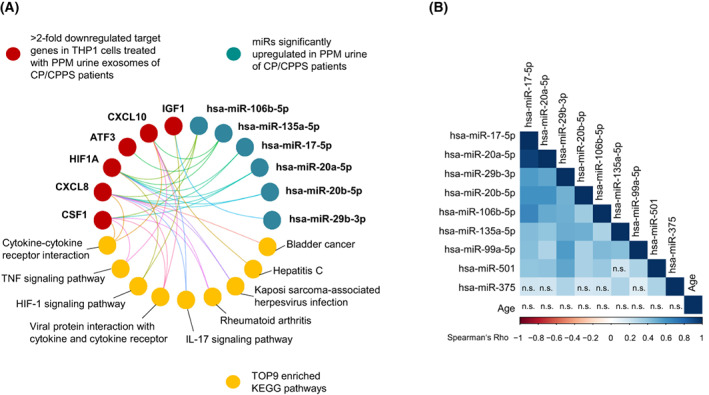
Analysis of microRNAs significantly associated with CP/CPPS and downregulated putative target genes with regard to potentially affected pathways. (A) The DOSE package of r/bioconductor was used to produce a Cnet plot, which visualizes the network of microRNAs, their target genes, and significantly enriched KEGG pathways. Six of nine microRNAs that were significantly upregulated in post‐prostatic‐massage (PPM) urine exosomes from CP/CPPS patients formed a network together with six putative target genes showing > 2‐fold downregulation in THP‐1 cells upon 24 h treatment with CP/CPPS‐derived PPM urine exosomes. (B) Nine microRNAs significantly upregulated in PPM urine exosomes extracted from CP/CPPS patients were analyzed by Spearman correlation analysis with regard to mutual correlation and correlation with age using TCGA dataset of 337 PCa specimens. Except for the cases indicated as not significant (n.s.), the expression of all detected microRNAs significantly correlated (Spearman's Rho > 0.3, two‐sided *P*‐values < 0.001). None of the microRNAs correlated with age (Spearman's Rho < 0.1, two‐sided *P*‐values > 0.1) (see also Table S6).

To understand the disease ontology of CP/CPPS type IIIb, we performed a DOSE analysis. Nine significantly upregulated microRNAs detected in PPM urine exosomes from CP/CPPS patients were analyzed together with the six target genes (*CSF1*, *CXCL8*, *CXCL10*, *IGF1*, *HIF1A*, and *ATF3*) showing significant downregulation in THP‐1 cells upon treatment with CP/CPPS‐derived PPM urine exosomes. Six of nine upregulated microRNAs formed a Cnet plot with six target genes, and nine KEGG pathways were identified as significantly enriched: “Bladder cancer,” “Hepatitis C," “Kaposi sarcoma‐associated herpes virus infection,” “Rheumatoid arthritis,” “IL‐17 signaling,” “Viral protein interaction with cytokine and cytokine receptor,” “HIF‐1 signaling pathway,” “TNF signaling pathway,” and “Cytokine‐cytokine receptor interaction” (Benjamini–Hochberg adjusted *P* < 0.01; Fig. 8A).

As our CP/CPPS patient group was older than the healthy control group (median age 50.9 years versus 31.4 years), we analyzed whether the expression of the nine microRNAs upregulated in PPM urine exosomes from CP/CPPS patients correlates with age. Therefore, we used the dataset of 337 PCa specimens from TCGA. We found that none of the microRNAs correlated with age (Spearman's Rho < 0.1, two‐sided *P* > 0.1; Fig. 8B; Table S6). Remarkably, a strong mutual positive correlation was found in PCa for all nine microRNAs (Spearman's Rho mostly 0.4–0.9, two‐sided *P* < 0.001; Fig. 8B; Table S6).

## 4 Discussion

Chronic inflammation is assumed to predispose the tissue to the development of cancer and promote all stages of tumorigenesis. Identifying the critical steps leading from inflammation to cancer initiation could provide points for cancer prevention and important markers for the diagnosis of early‐stage cancer. However, molecular studies in CP/CPPS patients are still scarce, and at present, it remains unclear whether CP/CPPS poses a carcinogenic risk for men. In this context, our present study aimed primarily at providing molecular evidence that CP/CPPS occurring frequently in men at an average age of 47.2 years (standard deviation 15.7) [10] may pose a risk factor for PCa development. We performed a broad spectrum of molecular experiments using liquid biopsies of CP/CPPS patients and healthy men and found a striking difference in CP/CPPS in terms of the mircoRNA profiles and epigenetic potential of exosomes in PPM urine, pointing to the dysregulation of PCa‐associated genes and pathways in CP/CPPS and the possibility of fostering the development of PCa.

Compared to “normal” urine, PPM urine may contain cells, organelles, and fluids originating from the prostate and, therefore, offer an ideal material for investigating CP/CPPS and overcoming the lack of tissue biopsies. Investigating PPM urine exosomes, we found nine significantly upregulated microRNAs in CP/CPPS, eight of which (hsa‐miR‐106b‐5p, hsa‐miR‐135a‐5p, hsa‐miR‐17‐5p, hsa‐miR‐20a‐5p, hsa‐miR‐20b‐5p, hsa‐miR‐29b‐3p, hsa‐miR‐375, and hsa‐miR‐501) were also found to be significantly upregulated in PCa when analyzing TCGA datasets comprising more than 300 PCa specimens. All microRNAs upregulated in CP/CPPS have been shown to play a crucial role in the development, progression, and/or metastasis of PCa or other cancer entities [40, 41, 42, 43, 44, 45, 46, 47]. MicroRNAs regulate the expression of their target genes at the post‐transcriptional level, and the upregulation of microRNAs expectedly implies a downregulation of their targets. However, the situation is complicated by the fact that one microRNA may target several genes, and one gene may be regulated by several microRNAs as shown in Fig. 8. Moreover, it is known that microRNAs may also indirectly activate the expression of genes by inhibiting intermediate suppressors. By analyzing the common putative target genes of at least four microRNAs upregulated in CP/CPPS, we detected 825 genes, which were most significantly enriched for GO terms “Regulation of neurogenesis,” “Positive regulation of cellular catabolic process,” and “Ras protein signal transduction.” The neurological aspect, particularly chronic pain, plays a central role in CP/CPPS, but is difficult to investigate due to the imprecision of pain localization and multifactorial etiology, and is still poorly understood. A recent study reported that a diverse set of dysregulated neuroinflammatory genes is found in the blood and urine of men with CP/CPPS [48]. We revealed an additional 37 factors potentially involved in CP/CPPS‐associated dysregulation of neurogenesis, which may build the molecular basis for chronic pain. Concerning catabolism, as is already known, the catabolic processes, such as the citric acid cycle, glycolysis, lipolysis, oxidative deamination, or muscle tissue breakdown, are part of the metabolism, and it is thought that tumors reprogram nutrient acquisition and metabolism pathways to meet the bioenergetic, biosynthetic, and redox demands of malignant cells [49]. Considering this, our results suggest that CP/CPPS may present a chronic condition with adverse effects on catabolism in the prostate. Furthermore, Ras is a proto‐oncogene and members of this family have been argued to drive PCa progression to a state of androgen hypersensitivity, particularly via their interplay with growth factors and androgen receptors [50]. Further, KEGG pathway analyses revealed that putative target genes of CP/CPPS‐specific upregulated microRNAs were significantly enriched for cancer‐associated pathways, such as “Prostate cancer,” “MAPK signaling pathway,” “FoxO signaling pathway,” and “Non‐small cell lung cancer.” Among others, these pathways contain several factors involved in the regulation of growth factors, cytokine–cytokine receptor interaction, apoptosis, cell cycle progression, cellular migration and invasion, and transcriptional regulation. MicroRNAs from urinary exosomes have already been suggested as alternative biomarkers in the differentiation of benign and malignant prostate diseases [51]. Our results suggest that microRNAs from PPM urine of CP/CPPS may be early prognostic biomarkers of malignant transformation of the prostate. However, considering the multifactorial complex disease situation in CP/CPPS, the causal relation between detected upregulated microRNAs and predictive targets, and the specific function of each of the latter should be clarified in further mechanistic studies.

Exosomes play a central role in cell‐to‐cell communication by transferring microRNAs into acceptor cells and altering the gene expression and function [31]. We found that PPM urine exosomes from men with CP/CPPS affected the polarization of THP‐1 monocytes and induced abnormal expression of several inflammatory genes known to be crucially involved in cancer‐associated inflammation, as well as the expression of many transcription factor‐encoding genes. Among inflammatory genes, the highest upregulation was observed for *CCR2* (sevenfold, encoding C‐C chemokine receptor type 2) and *CCL18* (fourfold, encoding C‐C motif chemokine ligand 18). Importantly, upregulation of CCR2 and its ligand CCL2 has also been found in PCa and associated with cancer advancement, metastasis, and relapse [52], and upregulation of CCL18 has been shown to correlate with malignant progression of PCa [53]. Among transcription factors, the highest upregulation was observed for *MYB* (ninefold, MYB proto‐oncogene), *HOXA5* (fivefold, encoding homeobox protein Hox‐A5), *JUNB* (fivefold, JunB proto‐oncogene), and *STAT2* (fourfold, encoding signal transducer and activator of transcription 2). In terms of PCa, MYB was recently shown to interact with androgen receptor and to promote castration resistance [54], whereas JUNB expression was increased in low‐grade PCa compared to normal prostate but downregulated in high‐grade samples [55]. HOXA5 and STAT2 are currently less researched in PCa. Our further analyses of the disease ontology in CP/CPPS (i.e., DOSE analyses) suggested a functional network between six microRNAs found to be significantly upregulated in PPM urine exosomes in CP/CPPS and their six putative target genes (*IGF1*, *CXCL10*, *ATF3*, *HIF1A*, *CXCL8*, and *CSF1*), which were found to be > 2‐fold downregulated in THP‐1 cells when treated with CP/CPPS‐originating urine exosomes. Here, several pathways involved in carcinogenesis and cancer‐associated viral infection as well as inflammatory and hypoxia‐related processes were potentially affected in CP/CPPS. While the specific role of *ATF3* (activating transcription factor 3) in the suppression of PCa with PTEN (phosphatase and tensin homolog) dysfunction is meanwhile well known [56, 57], the tumor suppressor function of *IGF1*, *CXCL10*, *HIF1A*, *CXCL8*, and *CSF1* in prostate carcinogenesis remains to be elucidated. However, analyzing TCGA data from > 300 PCa specimens, we found *IGF1*, *HIF1A*, *CXCL8*, and *CSF1* to be significantly downregulated in PCa in comparison to normal prostate.

In contrast to PPM urine exosomes, exosomes isolated from the blood serum of men with CP/CPPS did not differ from those isolated from healthy men in the quantities of exosome‐specific marker proteins CD81, CD63, CD9, and HSP70 or PCa‐specific microRNAs. Slightly increased PSA levels were detected in blood serum exosomes from CP/CPPS patients, although PSA signals on western blot were very weak. Two proteins elevated in PCa‐driven exosomes, namely FAS [58, 59] and survivin [60, 61], were tested in CP/CPPS‐driven blood serum exosomes but not detected. As men with CP/CPPS tend to have a sixfold greater history of cardiovascular disease [11], we analyzed the capacity of blood serum exosomes from CP/CPPS patients and healthy men to activate endothelial cells, i.e., to induce a proinflammatory and procoagulant state of the endothelial cells lining the lumen of blood vessels. Serum exosomes from both CP/CPPS and healthy men were not able to activate neither HUVEC nor primary endothelial cells within 24 and 48 h. These results suggest that CP/CPPS‐associated molecular changes may be limited to the local urogenital effects and are more pronounced in PPM urine.

We would like to point out that our study is the first of its kind in this research area and is limited by the relatively low number of CP/CPPS patients and healthy men investigated (*n* = 20 per group for expression analyses, and additional *n* = 5 for array and cell culture experiments). The number of used specimens in different experiments conformed to the availability of material, practicability of experiments, and financial circumstances. The used number was sufficient enough to find significant differences between the two groups but was insufficient for performing solid association and correlation studies between molecular and clinical parameters within CP/CPPS group. In addition, the heterogeneity of clinical phenotypes among CP/CPPS type IIIb patients and the absence of standardized markers for disease severity and progress presented a challenge for making individual prognoses. It is probable that not all CP/CPPS patients will develop PCa, and additional unfavorable conditions have to occur or accumulate during the course of life. As most CP/CPPS patients are 45–50 years of age, and PCa and BPH mostly occur much later in life at > 65 years of age, it is impossible at the moment to conclude whether our studied probands will develop PCa. Further follow‐up studies on a larger number of subjects and selected molecular factors, genome‐wide studies, and suitable model systems are needed to confirm our findings in detail.

## 5 Conclusions

Overall, our study provides novel insights into the molecular pathogenesis of CP/CPPS type IIIb and its possible link to PCa by showing a significant dysregulation of PCa‐associated microRNAs as well as specific inflammatory factors, transcription factors, and pathways. Moreover, we demonstrated that PPM urine of CP/CPPS patients has the potential as source for biomarker development.

## Conflict of interest

The authors declare no conflict of interest.

## Author contributions

US designed and supervised the study. LS performed experiments (isolation of exosomes, western blot, RT‐qPCR, miScript PCR Array, THP‐1 treatment, ICC, and RT^2^ Profiler PCR array). TD performed bioinformatic analyses and corresponding statistical analyses. EN supervised experiments in synovial tissue, HUVEC, and primary endothelial cells (IHC, ICC, RT‐qPCR, and ELISA). HY performed THP‐1 polarization and subsequent RT‐qPCR. AP and FW examined and treated the CP/CPPS patients, recruited them for the study, and took responsibility for the clinical aspects. H‐CS took responsibility for the andrological aspects. LS, TD, and US analyzed and interpreted the data. TD and US prepared the figures, and wrote and revised the manuscript. All authors read and approved the manuscript.

### Peer Review

The peer review history for this article is available at https://publons.com/publon/10.1002/1878‐0261.13329.

## Supporting information


**Fig. S1.** Transmission electron microscopy (TEM) analysis of blood serum exosomes and post‐prostatic‐massage (PPM) urine exosomes.
**Fig. S2.** REVERT™ total protein stain of serum exosome proteins, and detection and quantification of exosome marker proteins HSP70, CD81, CD63, and CD9 (two exemplary western blots are shown).
**Fig. S3.** Detection of the prostate‐specific antigen (PSA), fatty acid synthase (FAS), and survivin in blood serum exosomes.
**Fig. S4.** Validation of primer sets used for analysis of micro(mi)‐RNAs in exosomes.
**Fig. S5.** Comparison of miRNA levels in normal prostate and prostate cancer.
**Fig. S6.** miScript miRNA PCR array “Human prostate cancer” analysis in post‐prostatic‐massage (PPM) urine exosomes.
**Fig. S7.** Validation of the specificity of the anti‐CD81 antibody.
**Fig. S8.** Validation of primer sets used for RT‐qPCR analyses in THP‐1 cells.
**Fig. S9.** Polarization of THP‐1 cells in M1 and M2 macrophages.
**Fig. S10.** Treatment of THP‐1 cells with post‐prostatic‐massage (PPM) urine exosomes.
**Fig. S11.** Treatment of THP‐1 cells with blood serum exosomes.
**Fig. S12.** Treatment of HUVEC (human umbilical vein endothelial cells) with blood serum exosomes.
**Fig. S13.** Treatment of primary endothelial cells (ECs) with blood serum exosomes.
**Table S1.** Primary and secondary antibodies used for quantitative western blot, immunocytochemistry, and immunohistochemistry.
**Table S2.** Kits, assays, and controls used for miRNA analyses by RT‐qPCR.
**Table S3.** Arrays used for profiling of miRNAs in post‐prostatic‐massage (PPM) urine exosomes and gene expression in THP‐1 after treatment with PPM urine exosomes.
**Table S4.** Primer sets used for gene expression analyses by RT‐qPCR in THP‐1 cells treated with post‐prostatic‐massage urine exosomes, and in HUVEC and primary endothelial cells treated with blood serum exosomes.
**Table S5.** Enzyme‐linked immunosorbent assay kits used for protein analyses in HUVEC and primary endothelial cells treated with blood serum exosomes.
**Table S6.** Spearman's Rho correlation analysis between age and expression of microRNAs, which were significantly upregulated in post‐prostatic‐massage urine exosomes of CP/CPPS patients, by using the TCGA dataset (n = 337 PCa specimens).
**Table S7.** Results of the miSCript miRNA PCR array “Human Prostate Cancer” analysis of RNA isolated from post‐prostatic‐massage urine exosomes of CP/CPPS patients (n = 3) and healthy men (n = 3).
**Table S8.** Gene ontology enrichment analysis on 825 putative target genes of miRNAs*, which were significantly upregulated in post‐prostatic‐massage (PPM) urine exosomes of CP/CPPS patients.
**Table S9.** Hallmark gene sets enrichment analysis using Molecular Signature Database on 825 putative target genes of miRNAs, which were significantly upregulated in post‐prostatic‐massage (PPM) urine exosomes of CP/CPPS patients.
**Table S10.** Kyoto Encyclopedia of Genes and Genomes (KEGG) pathway enrichment analysis on 825 putative target genes of miRNAs, which were significantly upregulated in post‐prostatic‐massage (PPM) urine exosomes of CP/CPPS patients.
**Table S11.** RT^2^ Profiler™ PCR Array “Human Cancer Inflammation & Immunity Crosstalk” in untreated THP‐1 (M0) cells and THP‐1 treated for 24 h with post‐prostatic‐massage (PPM) urine exosomes of healthy men (n = 2) and CP/CPPS patients (n = 3).
**Table S12.** RT^2^ Profiler™ PCR Array “Human Transcription Factors” in untreated THP‐1 (M0) cells and THP‐1 treated for 24 h with post‐prostatic‐massage (PPM) urine exosomes of healthy men (n = 2) and CP/CPPS patients (n = 3).

## Data Availability

The data that support the findings of this study are available in the Supporting Information of this article (Figs S1–S13 and Tables S1–S12).
