# Supplementary material for: Post‐prostatic‐massage urine exosomes of men with chronic prostatitis/chronic pelvic pain syndrome carry prostate‐cancer‐typical microRNAs and activate proto‐oncogenes
Source: Mol Oncol. 2022 Nov 17;17(3):445–68. doi: 10.1002/1878-0261.13329 (PMC9980307; doi:10.1002/1878-0261.13329)
Supplement: Supplementary file 1 — Fig. S1. Transmission electron microscopy (TEM) analysis of blood serum exosomes and post‐prostatic‐massage (PPM) urine exosomes. Fig. S2. REVERT™ total protein stain of serum exosome proteins, and detection and quantification of exosome marker proteins HSP70, CD81, CD63, and CD9 (two exemplary western blots are shown). Fig. S3. Detection of the prostate‐specific antigen (PSA), fatty acid synthase (FAS), and survivin in blood serum exosomes. Fig. S4. Validation of primer sets used for analysis of micro(mi)‐RNAs in exosomes. Fig. S5. Comparison of miRNA levels in normal prostate and prostate cancer. Fig. S6. miScript miRNA PCR array “Human prostate cancer” analysis in post‐prostatic‐massage (PPM) urine exosomes. Fig. S7. Validation of the specificity of the anti‐CD81 antibody. Fig. S8. Validation of primer sets used for RT‐qPCR analyses in THP‐1 cells. Fig. S9. Polarization of THP‐1 cells in M1 and M2 macrophages. Fig. S10. Treatment of THP‐1 cells with post‐prostatic‐massage (PPM) urine exosomes. Fig. S11. Treatment of THP‐1 cells with blood serum exosomes. Fig. S12. Treatment of HUVEC (human umbilical vein endothelial cells) with blood serum exosomes. Fig. S13. Treatment of primary endothelial cells (ECs) with blood serum exosomes. Table S1. Primary and secondary antibodies used for quantitative western blot, immunocytochemistry, and immunohistochemistry. Table S2. Kits, assays, and controls used for miRNA analyses by RT‐qPCR. Table S3. Arrays used for profiling of miRNAs in post‐prostatic‐massage (PPM) urine exosomes and gene expression in THP‐1 after treatment with PPM urine exosomes. Table S4. Primer sets used for gene expression analyses by RT‐qPCR in THP‐1 cells treated with post‐prostatic‐massage urine exosomes, and in HUVEC and primary endothelial cells treated with blood serum exosomes. Table S5. Enzyme‐linked immunosorbent assay kits used for protein analyses in HUVEC and primary endothelial cells treated with blood serum exosomes. Table S6. Spearman's Rho [file MOL2-17-445-s001.pdf]

Supplementary Figures

Supplementary Figure 1 Transmission electron microscopy (TEM) analysis of blood serum exosomes and post-prostatic massage (PPM) urine exosomes. (A) Blood serum exosomes exhibited sizes ranging from circa 30 nm to 120 nm as shown in two exemplary images. **(B)** PPM urine exosomes showed a considerably lower number and exhibited sizes of circa 30 nm to 50 nm as shown in two exemplary images. Magnification and scales are indicated. Arrows show exemplary exosomes.

A

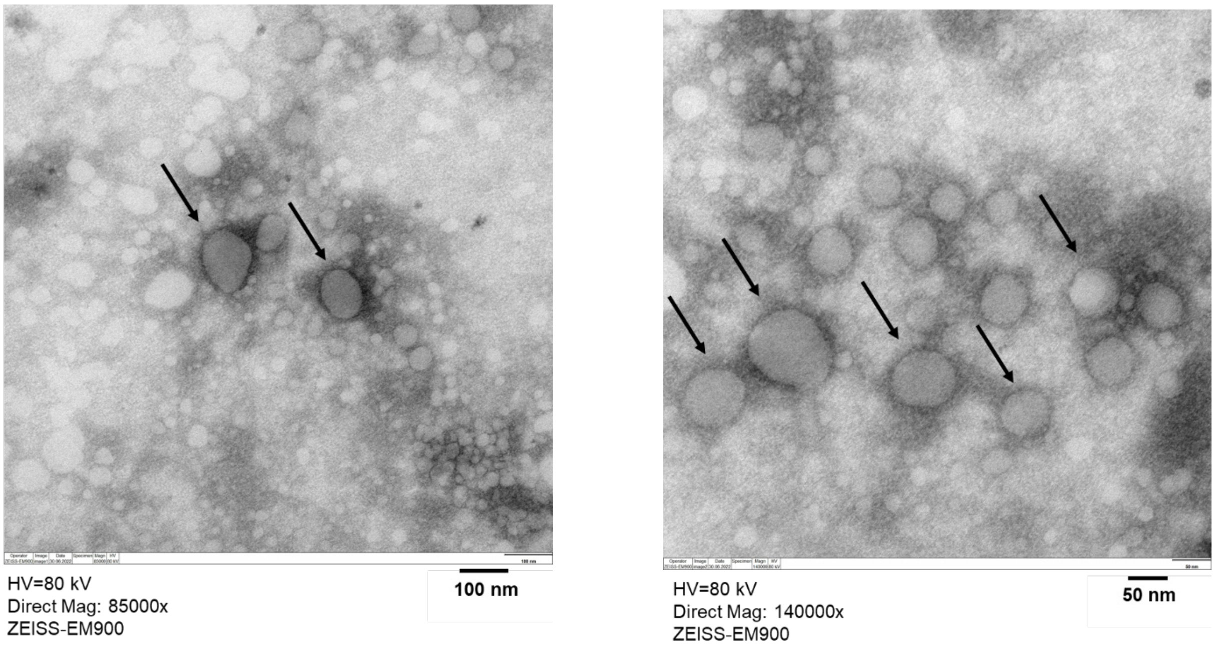

B

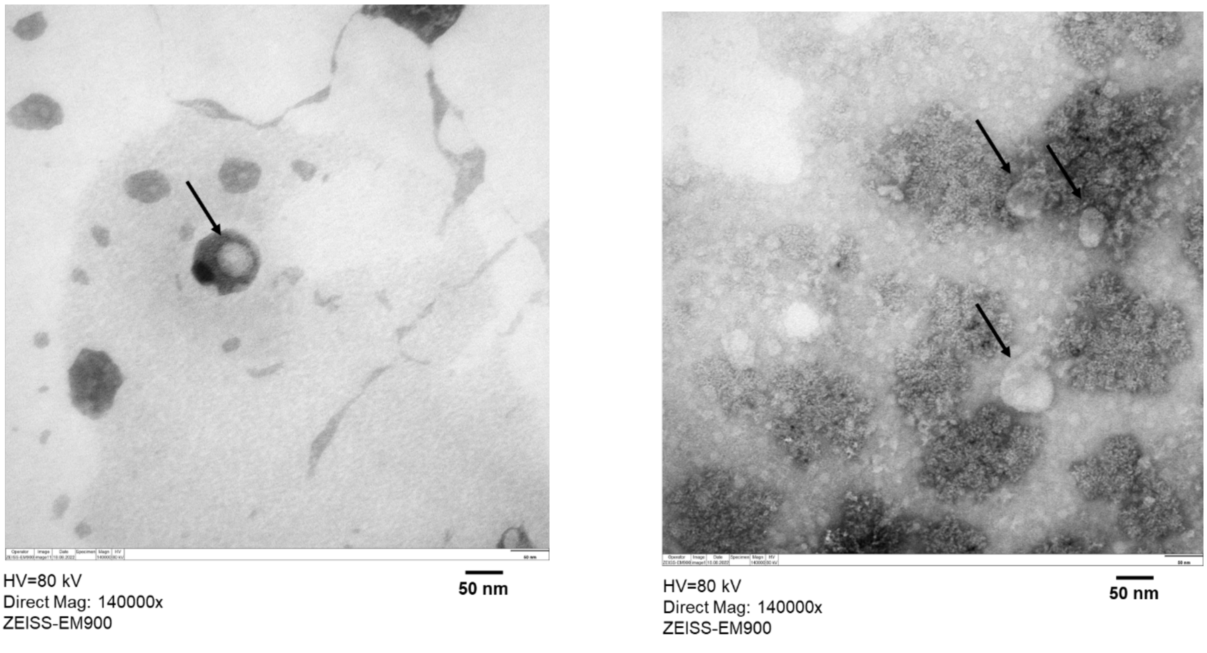

Supplementary Figure 2 REVERT™ total protein stain of serum exosome proteins, detection and quantification of exosome marker proteins HSP70, CD81, CD63 and CD9 (two exemplary western blots are shown). (A) Total protein isolated from blood serum exosomes of healthy men and patients suffering chronic prostatitis/chronic pelvic pain syndrome (CP/CPPS) was separated on sodium dodecyl sulfate polyacrylamid gel and visualized using LI-COR imaging system. As a positive (pos.) control, the Human Exosome Lysate Positive Protein Control (System Bioscience) was used at same amounts as the tested samples (40 µg). **(B and C)** The presence of exosome marker proteins HSP70 and CD81 **(B)**, CD63 and CD9 **(C)** was confirmed for all samples.

A

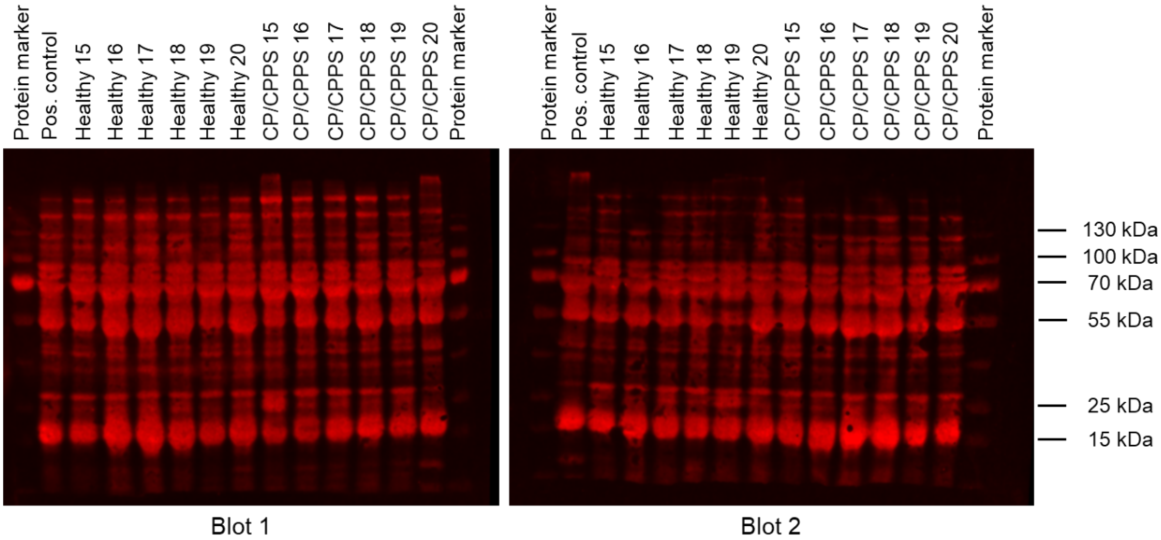

B

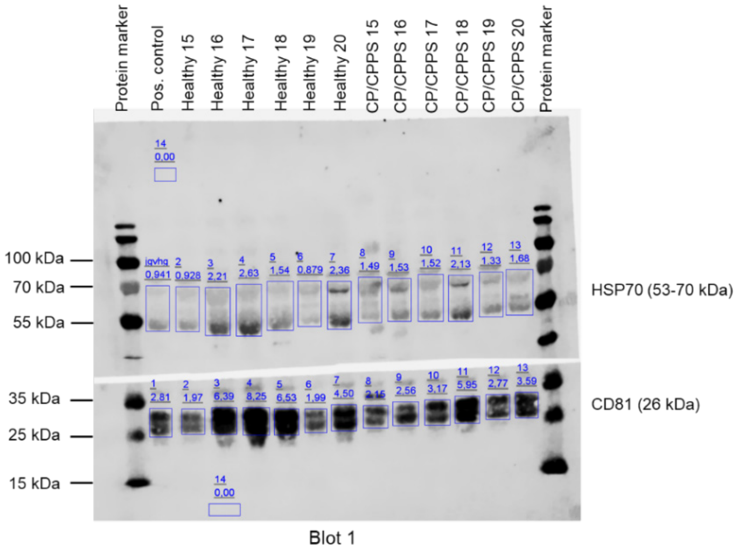

C

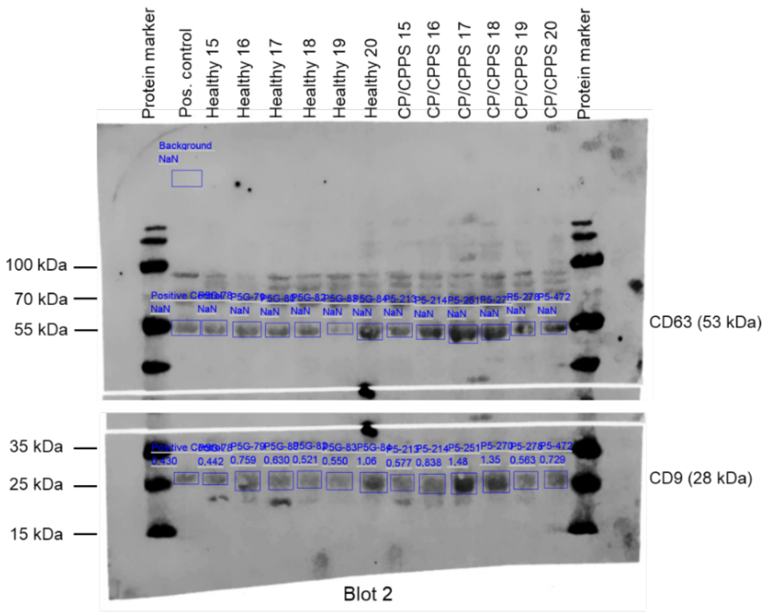

Supplementary Figure 3 Detection of the Prostate Specific Antigen (PSA), Fatty Acid Synthase (FAS) and Survivin in blood serum exosomes. (A) Total protein extracts isolated from serum exosomes of healthy men and CP/CPPS patients were analyzed for PSA (an exemplary western blot is shown) **(A.1).** PSA levels were found to be increased in serum exosomes of CP/CPPS patients in comparison to healthy men (p=0.0157, Mann-Whitney U test) **(A.2)**. FAS and Survivin were not detectable in serum exosomes of both healthy men and CP/CPPS patients (**B** and **C**; PC3 prostate cancer cell line was used as positive control).

A.1

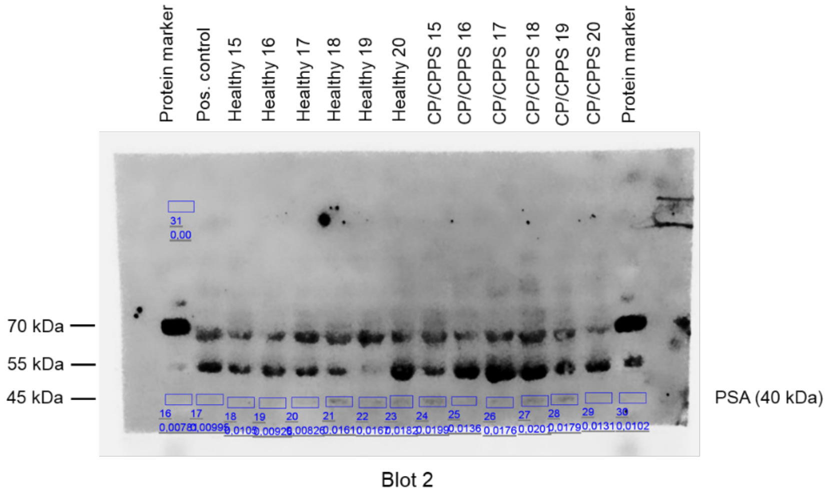

A.2

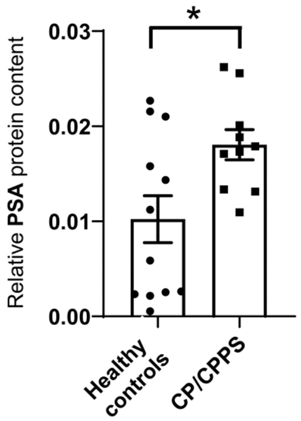

B

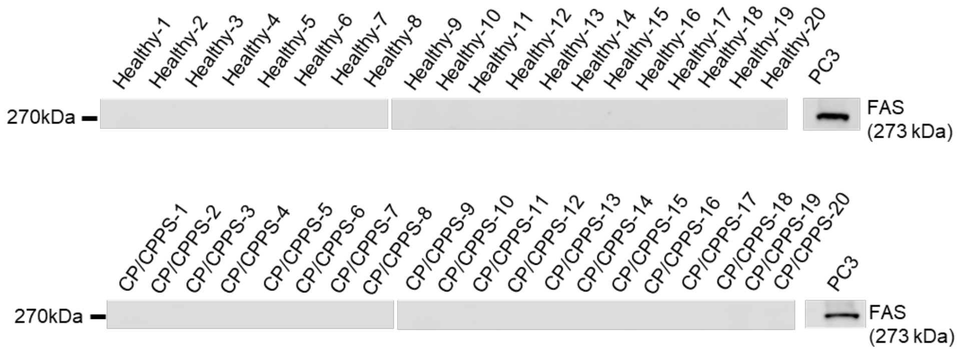

C

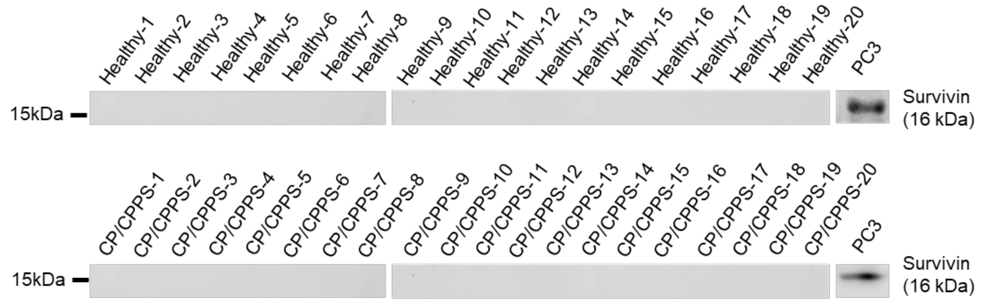

Supplementary Figure 4 Validation of primer sets used for analysis of micro(mi)-RNAs in exosomes. miRNAs known to be altered in prostate cancer (hsa-miRNA-141, hsa-miR-501, hsa-miR-532 and hsa-miR-375) were investigated. The positive control PCR primer set (Sigma-Aldrich, part of cat. no. MIRRT) was utilized as reference in quantification of miRNA levels. After reverse transcription (RT) of total exosome RNA in cDNA, a quantitative PCR (qPCR) was performed, and the products were checked on agarose gel.

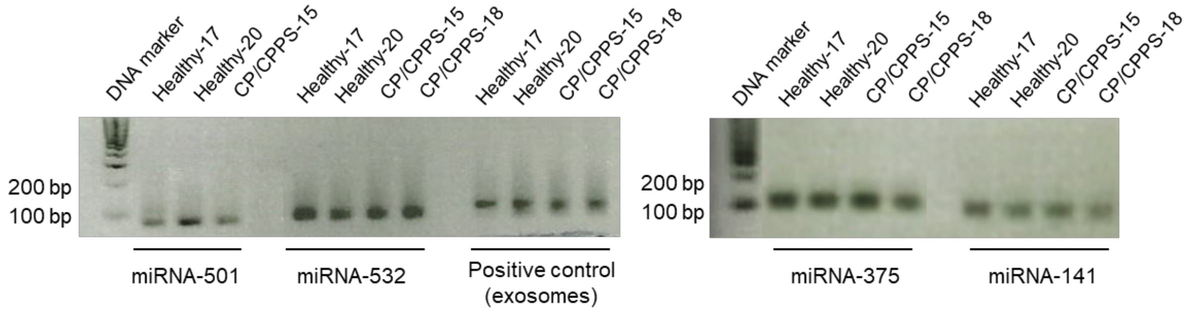

Supplementary Figure 5 Comparison of miRNA levels in normal prostate and prostate cancer. Expression levels of hsa-miR-141-3p (**A**), hsa-miR-532 (**B**), hsa-miR-99a-5p (**C**) and hsa-miR-22-3p (**D**) in normal prostate (NOR) and prostate cancer (PCa) were compared using the The Cancer Genome Atlas (TCGA) dataset (data were available for 35 NOR and 337 PCa). Mann-Whitney U test was used to compare the relative expression of microRNAs in two groups, and p-values <0.05 were considered as significant. Unit: fragments per kilobase per million mapped reads, FPKM.

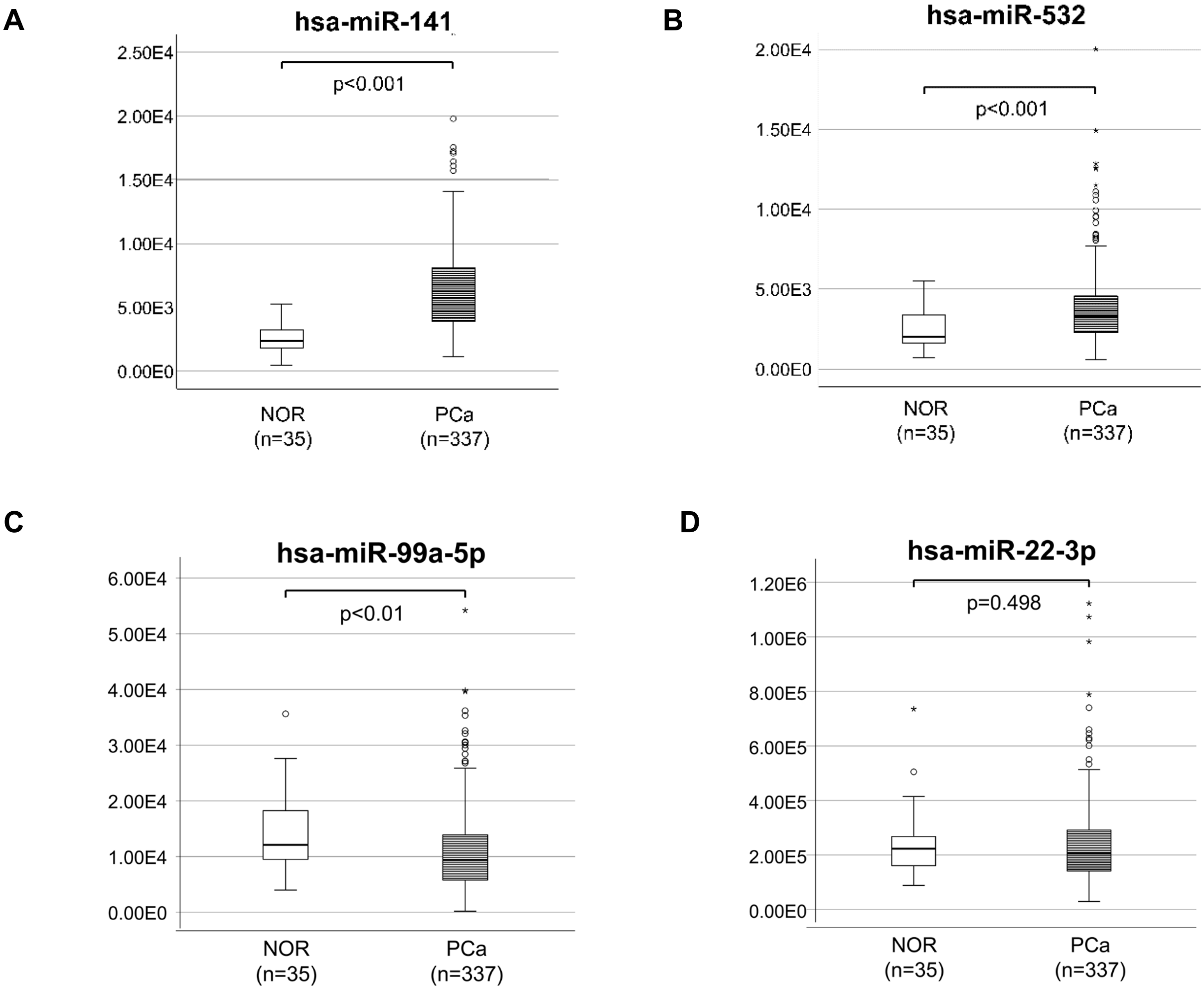

Supplementary Figure 6 miScript miRNA PCR array „Human prostate cancer“ analysis in post-prostatic-massage (PPM) urine exosomes. Total RNA samples isolated from PPM urine exosomes of patients with CP/CPPS (n=3) and healthy men (n=3) were analyzed using miScript miRNA PCR array „Human prostate cancer“ (Qiagen). The array comprises 84 miRNAs known to be crucially involved in prostate carcinogenesis. Log10 normalized scatter plots of detected miRNAs are shown together with the list of miRNAs exhibiting >2 fold change in CP/CPPS (mature miRNA identities, ID, are given) (**A-C**).

A

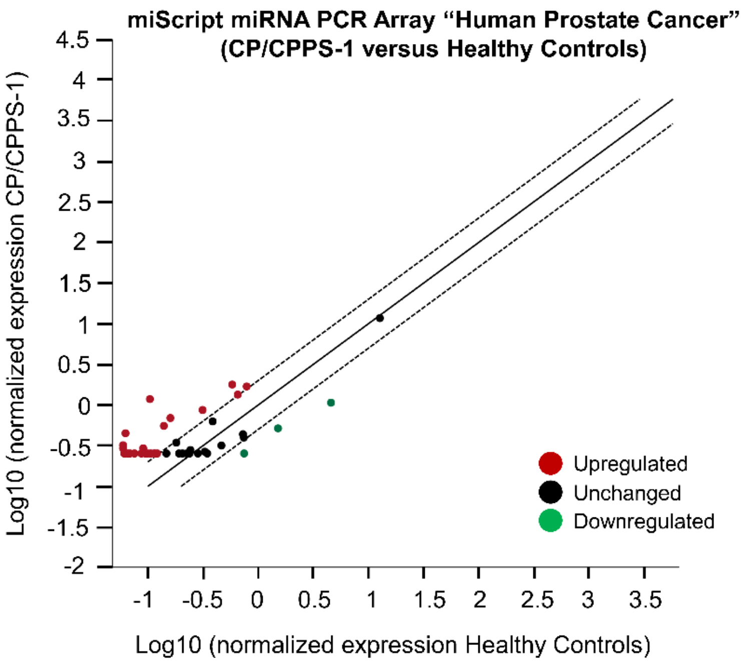

Mature miRNA ID	>2 fold regulation
hsa-let-7a-5p	2.39
hsa-let-7c-5p	3.87
hsa-miR-100-5p	3.07
hsa-miR-106b-5p	3.36
hsa-miR-125a-5p	2.15
hsa-miR-126-5p	2.58
hsa-miR-135a-5p	3.81
hsa-miR-141-3p	3.69
hsa-miR-143-3p	4.95
hsa-miR-145-5p	2.58
hsa-miR-148a-3p	-4.28
hsa-miR-15b-5p	2.22
hsa-miR-16-5p	2.77
hsa-miR-17-5p	3.83
hsa-miR-181b-5p	2.55
hsa-miR-194-5p	3.24
hsa-miR-200b-3p	2.06
hsa-miR-203a-3p	2.09
hsa-miR-205-5p	2.91
hsa-miR-20a-5p	2.58
hsa-miR-20b-5p	2.62
hsa-miR-22-3p	-2.94
hsa-miR-221-3p	4.08
hsa-miR-223-3p	7.18
hsa-miR-23b-3p	4.32
hsa-miR-29b-3p	3.96
hsa-miR-30c-5p	2.68
hsa-miR-3666	5.39
hsa-miR-425-5p	4.15
hsa-miR-99a-5p	2.22
hsa-miR-99b-5p	11.33

B

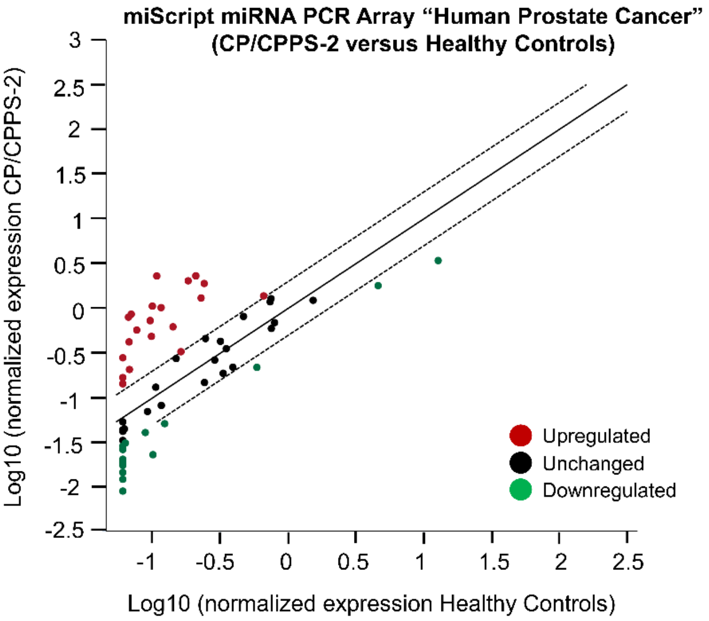

Mature miRNA ID	>2 fold regulation
hsa-let-7a-5p	21.55
hsa-let-7b-5p	5.74
hsa-let-7c-5p	12.08
hsa-let-7f-5p	4.67
hsa-miR-100-5p	-2.68
hsa-miR-106b-5p	7.51
hsa-miR-126-3p	-2.93
hsa-miR-128-3p	-3.42
hsa-miR-135a-5p	3.08
hsa-miR-135b-5p	-2.28
hsa-miR-141-3p	12.43
hsa-miR-148a-3p	-2.57
hsa-miR-17-5p	6.31
hsa-miR-181b-5p	-4.35
hsa-miR-182-5p	-2.08
hsa-miR-183-5p	-6.64
hsa-miR-200b-3p	2.10
hsa-miR-200c-3p	11.15
hsa-miR-203a-3p	-2.39
hsa-miR-205-5p	-2.16
hsa-miR-20a-5p	10.68
hsa-miR-20b-5p	4.98
hsa-miR-21-5p	-3.73
hsa-miR-221-3p	-2.01
hsa-miR-222-3p	-3.24
hsa-miR-23b-3p	2.03
hsa-miR-26a-5p	11.13
hsa-miR-26b-5p	7.92
hsa-miR-29b-3p	4.41
hsa-miR-30c-5p	7.63
hsa-miR-31-5p	-4.90
hsa-miR-32-5p	-4.10
hsa-miR-361-5p	-3.13
hsa-miR-374c-5p	2.39
hsa-miR-93-5p	2.80
hsa-miR-99a-5p	8.86

C

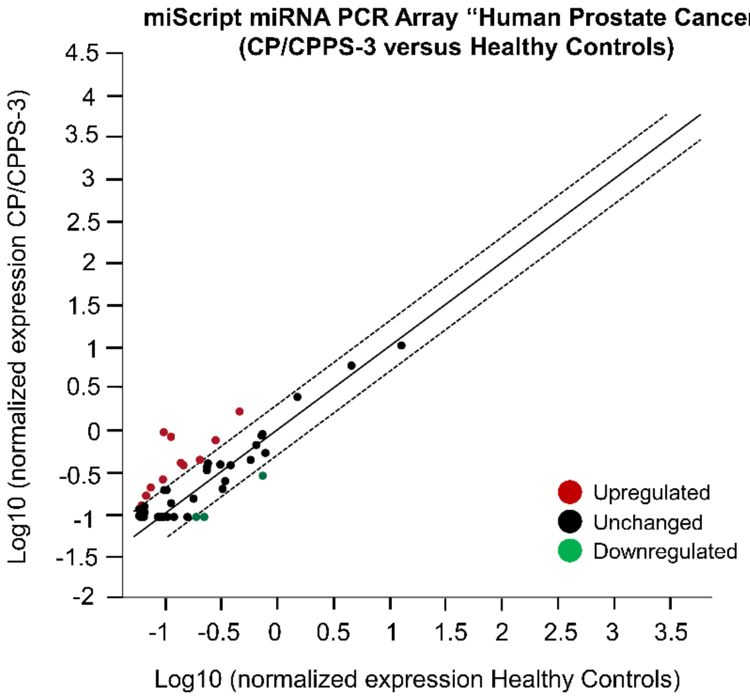

Mature miRNA ID	>2 fold regulation
hsa-let-7b-5p	-2.46
hsa-miR-101-3p	2.57
hsa-miR-106b-5p	2.72
hsa-miR-141-3p	2.40
hsa-miR-19b-3p	3.57
hsa-miR-20a-5p	9.53
hsa-miR-20b-5p	2.65
hsa-miR-22-3p	-2.62
hsa-miR-223-3p	2.01
hsa-miR-26a-5p	2.14
hsa-miR-29b-3p	2.89
hsa-miR-365b-3p	2.65
hsa-miR-455-5p	-2.09
hsa-miR-99a-5p	7.25

Supplementary Figure 7 Validation of the specificity of anti-CD81 antibody. Specificity of the anti-CD81 antibody (Abcam, cat. no. ab59477) was validated using synovial tissue from osteoarthritis patient by immunofluorescence (IF). (**A**) No signals were detectable in the negative (no primary antibody) control and isotype control. Tissue overview is shown in phase contrast (bottom left). IF of CD81 (green signal) showed specific signals mainly localized in the synovial lining layer and vessels (bottom, right, inset 1 and 2). Nuclei were stained blue with DAPI. (**B**) CD81 signals were localized in the synovial lining layer, specifically in the outer layers of the synovial lining layer (Inset 1), as well as close to vessel walls (Inset 2) (magnification in (A) 100-fold, in (B) 400-fold).

A

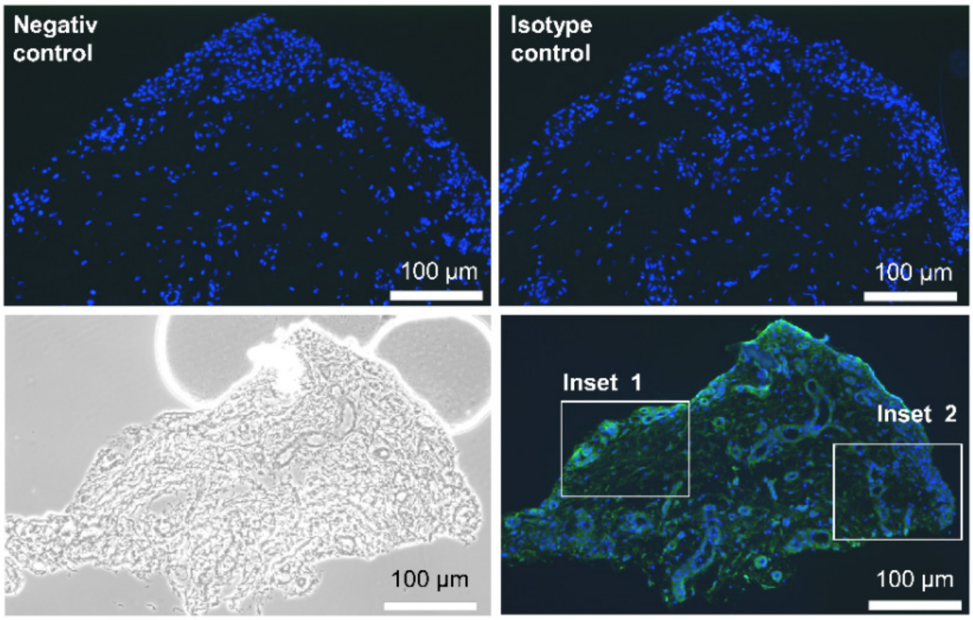

B

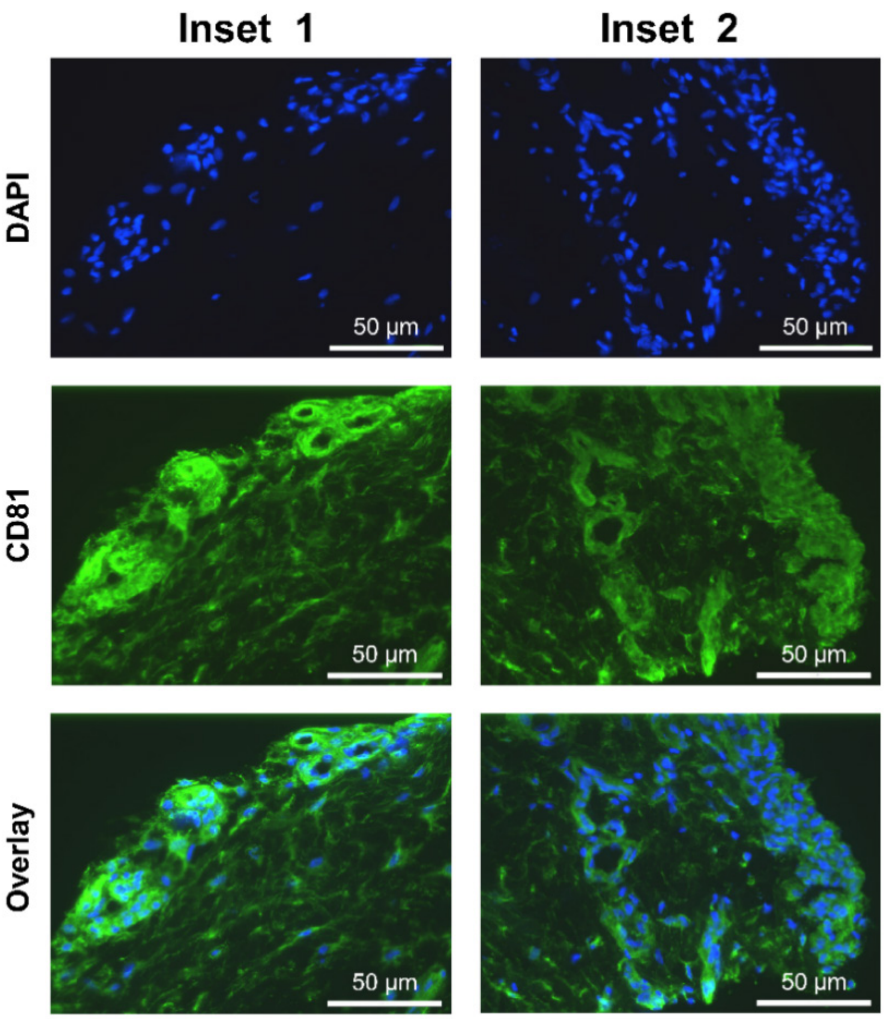

Supplementary Figure 8 Validation of primer sets used for RT-qPCR analyses in THP-1 cells. THP-1 cells in M0 state were treated with post-prostatic-massage urine exosomes of healthy men and CP/CPPS patients and analyzed for mRNA expression of the marker gene of M1 macrophages (*TNFα*, tumor necrosis factor alpha) and M2 macrophages (*CCL22*, CC-chemokine ligand 22). Expression of *GAPDH* (glyceraldehyde-3-phosphate dehydrogenase) was used as reference. Expected RT-qPCR product sizes were confirmed on agarose gel.

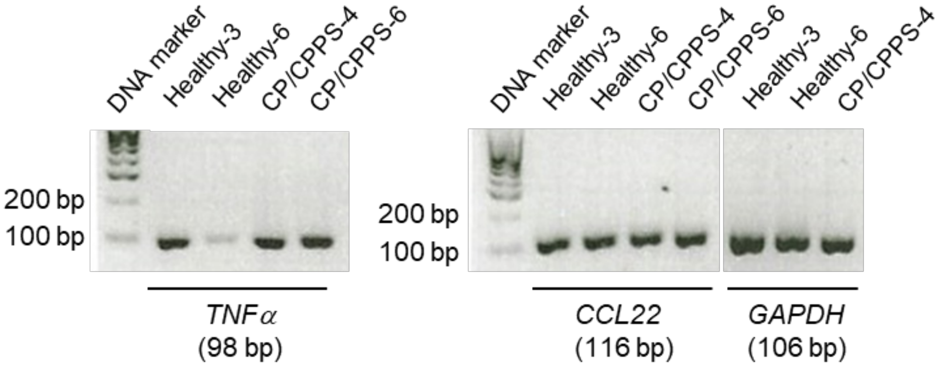

Supplementary Figure 9 Polarization of THP-1 cells in M1 and M2 macrophages. THP-1 cells at M0 state (naive macrophage-like state) were treated for 24 and 48 hours with interferon gamma and lipopolysaccharide for M1 polarization, and with interleukin-4 and interleukin-13 for M2 polarization. Polarization state was confirmed based on *TNFα* (M1 marker) (**A**) and *CCL22* (M2 marker) (**B**) expression (Student's t-test, p-values are given).

A

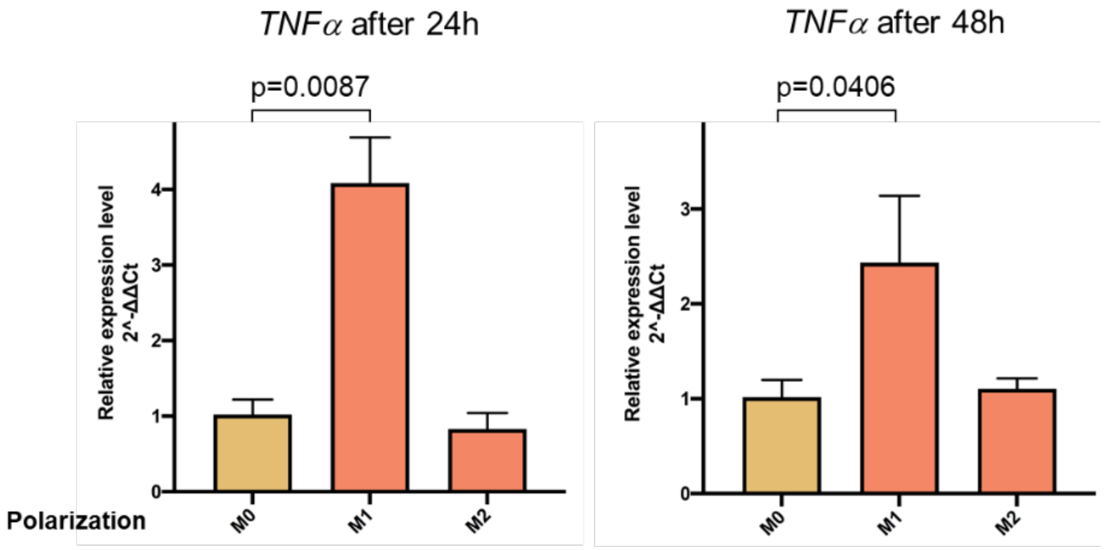

B

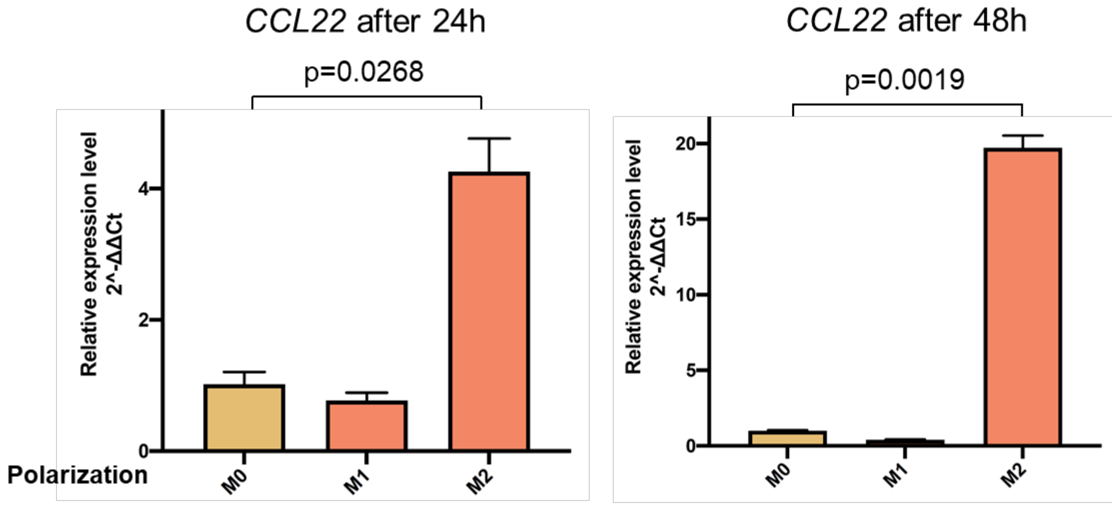

Supplementary Figure 10 Treatment of THP-1 cells with post-prostatic-massage (PPM) urine exosomes. THP-1 cells were treated at M0 state with PPM urine exosomes of healthy men and CP/CPPS patients for 48 h, and the intrusion of exosomes into THP-1 cells was analyzed by IF using CD81 antibody (Abcam, cat. no. ab59477, green signals). CD81 is an exosome-specific tetraspanin. CD81 signals within THP-1 cells after 48 h were weaker than detected after 24h treatment (shown in Figure 4). CD81 and CD81-Hoechst-overlay images are shown (Hoechst dye: blue fluorescent DNA stain).

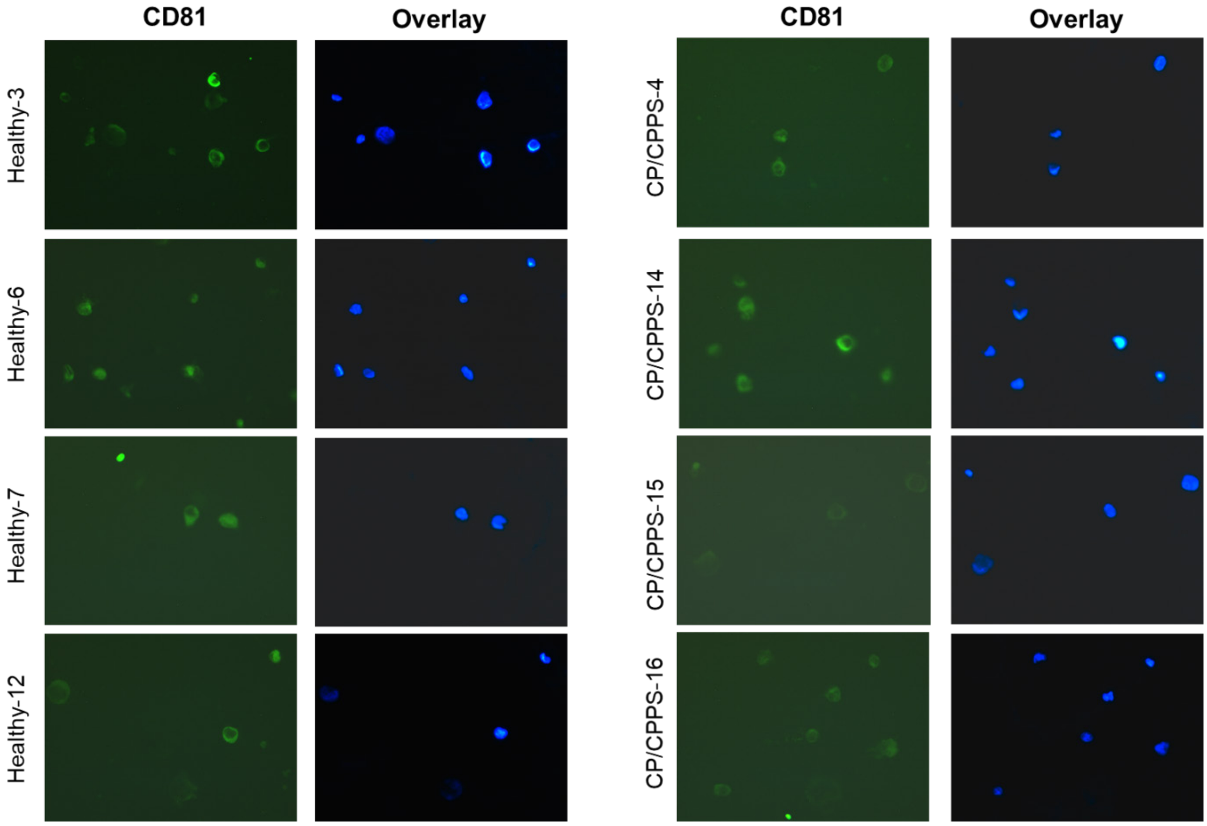

Supplementary Figure 11 Treatment of THP-1 cells with blood serum exosomes. THP-1 cells were treated at M0 state with blood serum exosomes of healthy men (**A**) and CP/CPPS patients (**B**) for 24 h, and the intrusion of exosomes into THP-1 cells was analyzed by IF using CD81 antibody (Abcam, cat. no. ab59477, green signals). CD81 signals documented after 24 h treatment of THP-1 cells with serum exosomes were noticeably weaker than after 24 h treatment with post-prostatic-massage urine exosomes (shown in Figure 4). Untreated THP-1 cells (M0) and cells incubated solely with the secondary antibody (no primary control) served as negative controls.

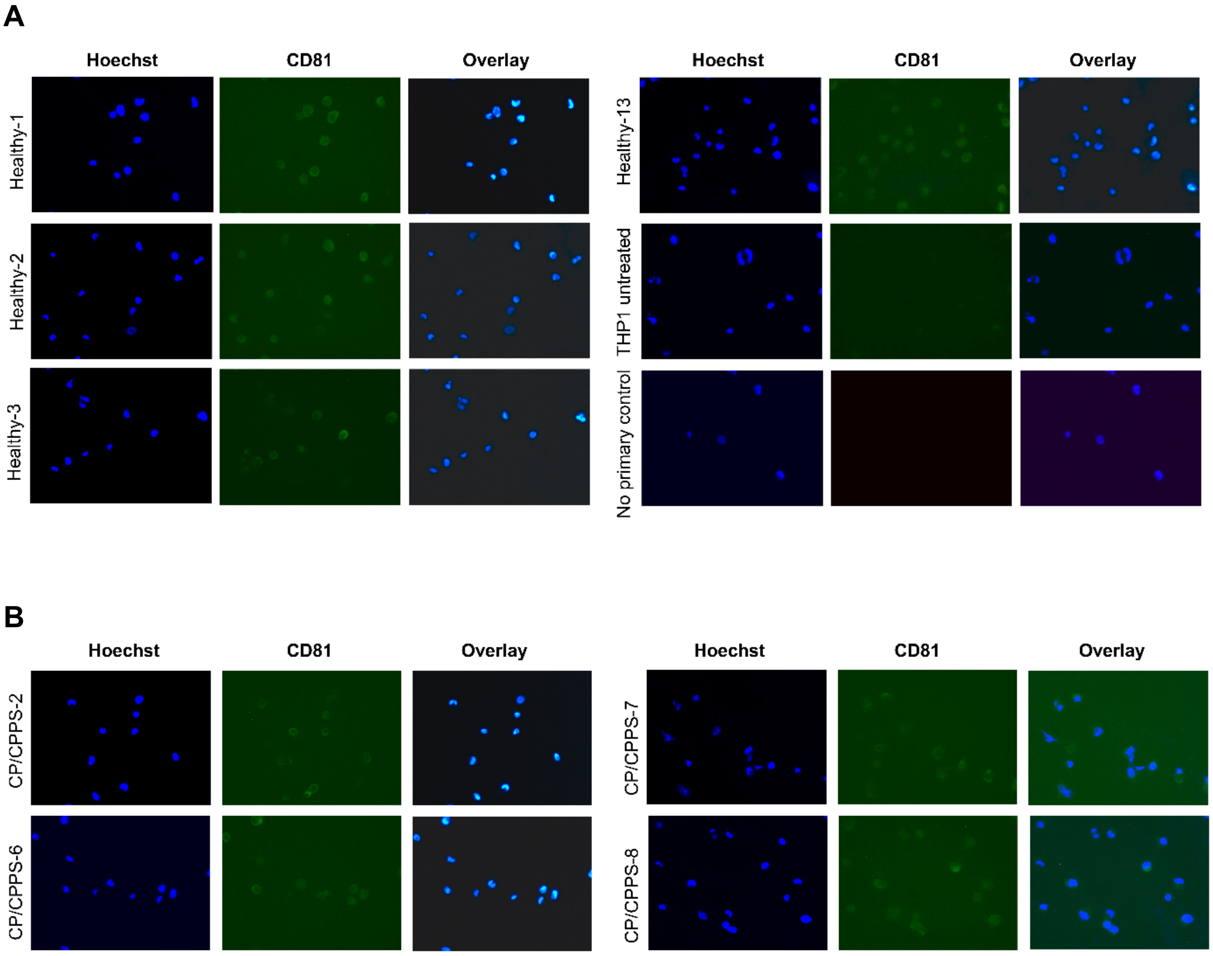

Supplementary Figure 12 Treatment of HUVEC (human umbilical vein endothelial cells) with blood serum exosomes. HUVEC were treated with blood serum exosomes of healthy men and CP/CPPS patients for 24 and 48 hours. Activation status of HUVEC was evaluated based on expression of Activin A (**A**), Interleukin-6 (IL-6) (**B**), and Monocyte Chemitactic Protein 1 (MCP1) (**C**) by Enzyme-linked Immunosorbent Assay (ELISA). HUVEC treated with ddH_2O and TNFα for 24 and 48 hours served as negative and positive controls, respectively. Treatment with blood serum exosomes did not affect the expression of endothelial cell activation markers in HUVEC (Fisher's exact test, p-values >0.05 were considered as not significant, n.s.).

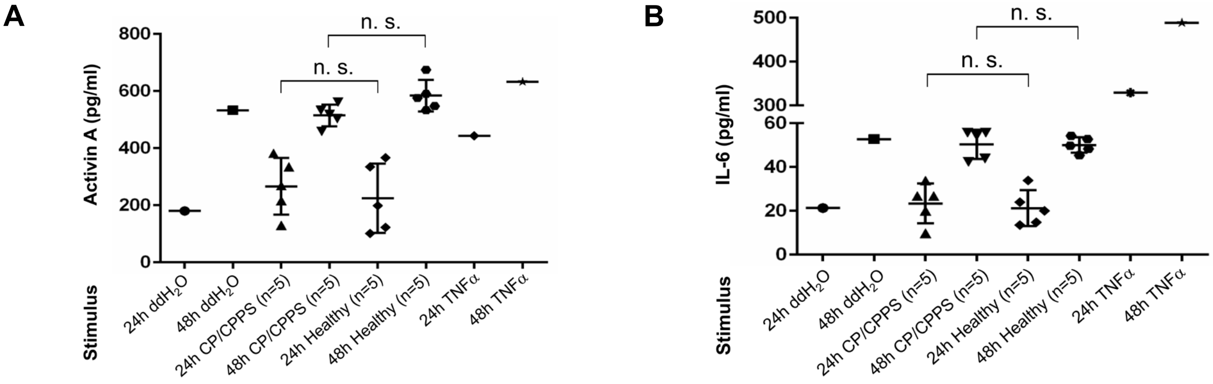

C

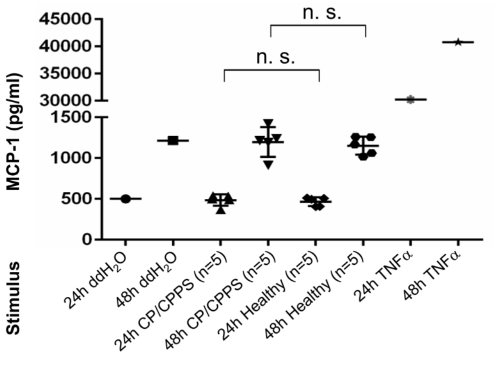

Supplementary Figure 13 Treatment of primary endothelial cells (ECs) with blood serum exosomes. Primary endothelial cells isolated from varicose veins were treated with blood serum exosomes of healthy men and CP/CPPS patients for 24 hours. Activation status of endothelial cells was analyzed based on protein expression of Activin A, IL-6 and MCP1 (**A.1-A.3**) by ELISA, and based on mRNA expression of *SELE* (Selectin E), *SELP* (Selectin P), *VCAM-1* (Vascular Cell Adhesion Molecule 1) and *ICAM-1* (Intercellular Adhesion Molecule 1) (**B.1-B.4**) by RT-qPCR. ECs treated with ddH_2O and TNFα for 24 hours served as negative and positive controls, respectively. Treatment with blood serum exosomes did not affect the expression of endothelial cell activation markers in ECs (Fisher's exact test, p-values >0.05 were considered as not significant, n.s.).

A.1

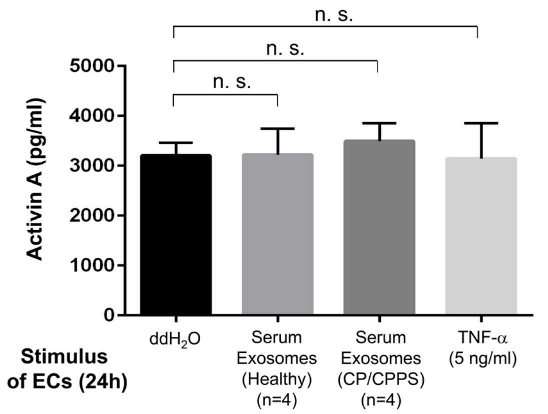

A.2

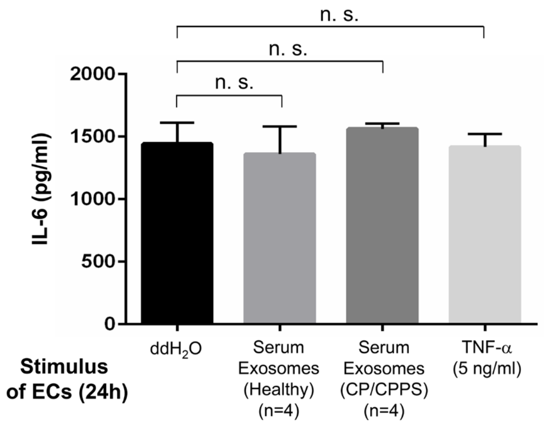

A.3

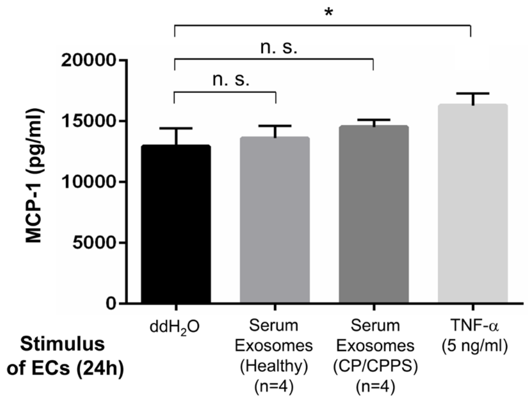

B.1

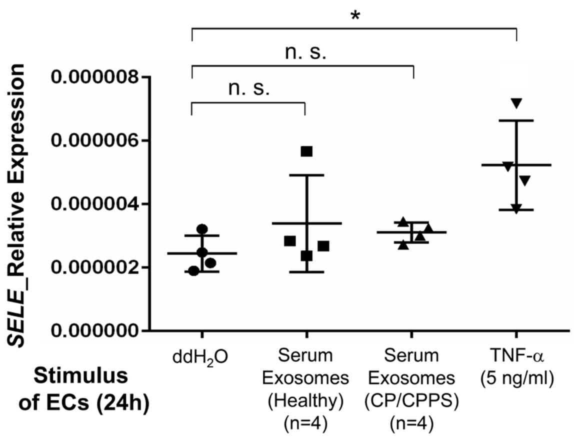
*
n. s.
n. s.
SELE_Relative Expression
0.000008
0.000006
0.000004
0.000002
0.000000
Stimulus of ECs (24h)
ddH2O
Serum Exosomes (Healthy) (n=4)
Serum Exosomes (CP/CPPS) (n=4)
TNF-α (5 ng/ml)

B.2

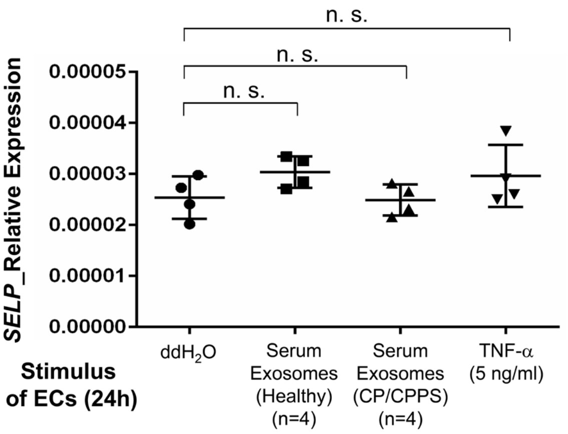
n. s.
n. s.
n. s.
SELP_Relative Expression
0.00005
0.00004
0.00003
0.00002
0.00001
0.00000
Stimulus of ECs (24h)
ddH2O
Serum Exosomes (Healthy) (n=4)
Serum Exosomes (CP/CPPS) (n=4)
TNF-α (5 ng/ml)

B.3

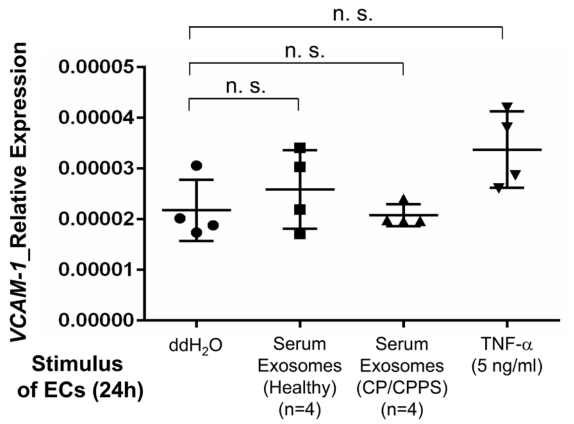
n. s.
n. s.
n. s.
VCAM-1_Relative Expression
0.00005
0.00004
0.00003
0.00002
0.00001
0.00000
Stimulus of ECs (24h)
ddH2O
Serum Exosomes (Healthy) (n=4)
Serum Exosomes (CP/CPPS) (n=4)
TNF-α (5 ng/ml)

B.4

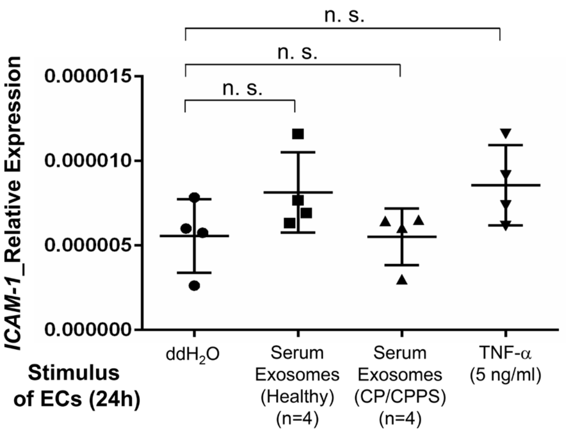
n. s.
n. s.
n. s.
ICAM-1_Relative Expression
0.000015
0.000010
0.000005
0.000000
Stimulus of ECs (24h)
ddH2O
Serum Exosomes (Healthy) (n=4)
Serum Exosomes (CP/CPPS) (n=4)
TNF-α (5 ng/ml)

Suppmenetary Table 1 Primary and secondary antibodies used for quantitative western blot, immunocytochemistry and immunohistochemistry

Antibody	Species	Dilution	Molecular weight	Manufacturer / Catalogue number
Anti-CD63 (qWB)	Rabbit	1:1500	~53 kDa	System Biosciences / EXOAB-CD63A-1
Anti-CD9 (qWB)	Rabbit	1:750	~28 kDa	System Biosciences / EXOAB-CD9A-1
Anti-CD81 (qWB)	Rabbit	1:1000	~26 kDa	System Biosciences / EXOAB-CD81A-1
Anti-HSP70 (qWB)	Rabbit	1:1000	~53-70 kDa	System Biosciences / EXOAB-Hsp70A-1
Anti-PSA (qWB)	Mouse	1:1000	~40 kDa	Thermo Fisher Scientific / MA5-17108
Anti-FAS (qWB)	Rabbit	1:750	~273 kDa	Abcam / ab128856
Anti-Survivin (qWB)	Rabbit	1:1000	~16 kDa	Abcam / ab76424
Anti-rabbit 680RD (qWB)	Goat	1:10000	-	LI-COR / 926-68073
Anti-mouse 800CW (qWB)	Goat	1:10000	-	LI-COR / 926-32210
Anti-CD81 (ICC, IHC)	Mouse	1:1000	~26 kDa	Abcam / ab59477
anti-Mouse IgG Alexa Fluor 488 (ICC)	Goat	1:10000	-	Thermo Fisher Scientific / A-28175
Hoechst 33342 (ICC)	-	1:500	-	Thermo Fisher Scientific / H-3570
anti-Mouse IgG Alexa Fluor 488 (ICC, IHC)	Goat	1:500	-	Thermo Fisher Scientific / A-11001
Anti-human IgG2a (ICC, IHC)	Mouse	1:250	-	BD Biosciences / 550339

Abbreviations: qWB: quantitative western blot; ICC: immunocytochemistry; IHC: immunohistochemitry; kDa: Kilodalton; CD63: Tetraspanin-30; CD9: Tetraspanin-29; CD81: Tetraspanin-28; HSP70: Heat shock 70kDa protein 4; PSA: prostate specific antigen; FAS: Fatty Acid Synthase.

Supplementary Table 2 Kits, assays and controls used for miRNA analyses by RT-qPCR

miRNA qPCR Assays	Mature Sequence	Melting point	Manufacturer / Catalogue number
MystiCq microRNA qPCR Assay Primer hsa-miR-141-5p (10 mg/ml)	CAUCUUCCAGUAC AGUGUUGGA	76 °C	Sigma-Aldrich Co. / MIRAP00173
MystiCq microRNA qPCR Assay Primer hsa-miR-375 (10 mg/ml)	UUUGUUCGUUCG GCUCGCGUGA	77.5 °C	Sigma-Aldrich Co. / MIRAP00360
MystiCq microRNA qPCR Assay Primer hsa-miR-532-3p (10 mg/ml)	CCUCCCACACCCA AGGCUUGCA	76.5 °C	Sigma-Aldrich Co. / MIRAP00515
MystiCq microRNA qPCR Assay Primer hsa-miR-501-3p (10 mg/ml)	AAUGCACCCGGG CAAGGAUUCU	76.5 °C	Sigma-Aldrich Co. / MIRAP00446
MystiCq microRNA Universal PCR Primer (10 mg/ml)	Not denoted	Not denoted	Sigma-Aldrich Co. / MIRUP
Human Positive Control Primer (10 mg/ml)	Not denoted	Not denoted	Sigma-Aldrich Co. / Part of MIRRT

Abbreviations: miRNA: microRNA; RT-qPCR: reverse transcription of RNA in cDNA followed by quantitative polymerase chain reaction.

Supplementary Table 3 Arrays used for profiling of miRNAs in post-prostatic massage (PPM) urine exosomes and of gene expression in THP-1 after treatment with PPM urine exosomes

Array name	Description	Manufacturer / Catalog number
miScript miRNA PCR Array „Human Prostate Cancer“	Array comprises 84 miRNAs known to be crucially involved in prostate cancer progression and development	Qiagen / MIHS-112Z
RT^2 Profiler „Human Cancer Inflammation and Immunity Crosstalk“	Array comprises 84 genes known to be crucially involved in human cancer inflammation and immunity crosstalk	Qiagen / PAHS-181Z
RT^2 Profiler „Human Transcription Factors“	Array comprises 84 genes encoding human transcription factors	Qiagen / PAHS-075Z

Supplementary Table 4 Primer sets used for gene expression analyses by RT-qPCR in THP-1 cells treated with post-prostatic massage urine exosomes, and in HUVEC and primary endothelial cells treated with blood serum exosomes

Gene	Primer (5‘-3‘) forward and reverse, Used Kits	Annealing T	Amplicon size (base pairs, bp)
TNFα	GTAGCCCATGTTGTAGCAAACC<br>TCTCTCAGCTCCACGCCATT	60°C	98 bp
CCL22	CGCGTGGTGAAACACTTCTAC<br>ATCTTCACCCAGGGCACTCT	60°C	116 bp
GAPDH	GCAAATTCCATGGCACCGT<br>TCGCCCCACTTGATTTTGG	60°C	106 bp
SELE	QT00015358 Hs_SELE_1_SG<br>QuantiTect Primer Assay	55°C	96 bp
SELP	QT00012516 HS_SELP_1_SG<br>QuantiTect Primer Assay	55°C	106 bp
VCAM-1	QT00018347 Hs_VCAM1_1_SG<br>QuantiTect Primer Assay	55°C	106 bp
ICAM-1	QT00074900 Hs_ICAM1_1_SG<br>QuantiTect Primer Assay	55°C	84 bp

Abbreviations: TNFα: Tumor necrosis factor alpha; CCL22: C-C motif chemokine ligand 22; GAPDH: Glyceraldehyde-3-phosphate dehydrogenase; SELE: Selectin E; SELP: Selectin P; VCAM-1: Vascular cell adhesion molecule 1; ICAM-1: Intercellular adhesion molecule 1.

Supplementary Table 5 Enzyme-linked Immunosorbent Assay kits used for protein analyses in HUVEC and primary endothelial cells treated with blood serum exosomes

Analyzed protein	Kit name	Manufacturer / Catalog number
Activin A	Human/Mouse/Rat Activin A Quantikine ELISA Kit	R&D/Bio-techne<br>DAC00B
IL-6	Human IL-6 Quantikine ELISA Kit	R&D/Bio-techne<br>D6050
MCP-1	Human CCL2/MCP-1 Quantikine ELISA Kit	R&D/Bio-techne<br>DCP00

Abbreviations: ELISA: enzyme-linked immunosorbent assay; IL-6: interleukin 6; MCP-1: monocyte chemotactic protein 1.

Supplementary Table 6 Spearman's rho correlation analysis between age and expression of microRNAs, which were significantly upregulated in post-prostatic-massage urine exosomes of CP/CPPS patients, by using TCGA dataset (n=337 PCa specimens)

**Spearman's correlation analysis**		Age	hsa-miR-106b-5p	hsa-miR-135a-5p	hsa-miR-17-5p	hsa-miR-20a-5p	hsa-miR-20b-5p	hsa-miR-22-3p	hsa-miR-29b-3p	hsa-miR-375	hsa-miR-501	hsa-miR-99a
Age ##	Rho###	1.000	0.088	-0.011	0.004	0.021	0.016	-0.081	0.037	0.024	0.076	-0.054
	p-value#		0.107	0.847	0.943	0.705	0.772	0.138	0.495	0.656	0.166	0.320
hsa-miR-106b-5p	Rho###	0.088	1.000	.311**	.654**	.508**	.359**	.378**	.489**	.191**	.377**	.444**
	p-value#	0.107		0.000	0.000	0.000	0.000	0.000	0.000	0.000	0.000	0.000
hsa-miR-135a-5p	Rho###	-0.011	.311**	1.000	.431**	.400**	.482**	.290**	.495**	.301**	.200**	.425**
	p-value#	0.847	0.000		0.000	0.000	0.000	0.000	0.000	0.000	0.000	0.000
hsa-miR-17-5p	Rho###	0.004	.654**	.431**	1.000	.940**	.608**	.352**	.563**	.200**	.399**	.429**
	p-value#	0.943	0.000	0.000		0.000	0.000	0.000	0.000	0.000	0.000	0.000
hsa-miR-20a-5p	Rho###	0.021	.508**	.400**	.940**	1.000	.608**	.272**	.522**	.197**	.403**	.304**
	p-value#	0.705	0.000	0.000	0.000		0.000	0.000	0.000	0.000	0.000	0.000
hsa-miR-20b-5p	Rho###	0.016	.359**	.482**	.608**	.608**	1.000	.254**	.477**	.188**	.321**	.325**
	p-value#	0.772	0.000	0.000	0.000	0.000		0.000	0.000	0.001	0.000	0.000
hsa-miR-22-3p	Rho###	-0.081	.378**	.290**	.352**	.272**	.254**	1.000	.611**	.252**	.632**	.654**
	p-value#	0.138	0.000	0.000	0.000	0.000	0.000		0.000	0.000	0.000	0.000
hsa-miR-29b-3p	Rho###	0.037	.489**	.495**	.563**	.522**	.477**	.611**	1.000	.301**	.528**	.565**
	p-value#	0.495	0.000	0.000	0.000	0.000	0.000	0.000		0.000	0.000	0.000
hsa-miR-375	Rho###	0.024	.191**	.301**	.200**	.197**	.188**	.252**	.301**	1.000	.311**	.136
	p-value#	0.656	0.000	0.000	0.000	0.000	0.001	0.000	0.000		0.000	0.013
hsa-miR-501	Rho###	0.076	.377**	.200**	.399**	.403**	.321**	.632**	.528**	.311**	1.000	.320**
	p-value#	0.166	0.000	0.000	0.000	0.000	0.000	0.000	0.000	0.000		0.000
hsa-miR-99a	Rho###	-0.054	.444**	.425**	.429**	.304**	.325**	.654**	.565**	.136	.320**	1.000
	p-value#	0.320	0.000	0.000	0.000	0.000	0.000	0.000	0.000	0.013	0.000	

Abbreviations and codes: CP/CPPS: chronic prostatitis chronic pelvic pain syndrome; TCGA: The Cancer Genome Atlas; ## Age of prostate cancer patients listed in TCGA was used for correlation of microRNA expression and age of men; ### Spearman's Rho; # 2-sided p-value; ** significant correlations.

Supplementary Table 7 Results of the miSCript miRNA PCR array „Human Prostate Cancer“ analysis of RNA isolated from post-prostatic-massage urine exosomes of CP/CPPS patients (n=3) and healthy men (n=3)

Mature miRNA IDs Human Prostate Cancer	Up-Down Fold Regulation in CP/CPPS (n=3) (comparison to healthy men, n=3)			p-value
	CP/CPPS-1	CP/CPPS-2	CP/CPPS-3	CP/CPPS versus Healthy men
hsa-let-7a-5p	2.39	21.55	-1.16	
hsa-let-7b-5p	1.13	5.74	-2.46	
hsa-let-7c-5p	3.87	12.08	1.40	
hsa-let-7f-5p	n. d.	4.67	n. d.	
hsa-miR-100-5p	3.07	-2.68	-1.33	
hsa-miR-101-3p	1.72	1.85	2.57	
hsa-miR-106b-5p	3.36	7.51	2.72	0.03
hsa-miR-125a-5p	2.15	-1.15	-1.48	
hsa-miR-125b-5p	-1.86	1.72	1.21	
hsa-miR-126-3p	n. d.	-2.93	n. d.	
hsa-miR-126-5p	2.58	n. d.	-1.07	
hsa-miR-128-3p	n. d.	-3.42	n. d.	
hsa-miR-133a-3p	n. d.	n. d.	n. d.	
hsa-miR-135a-5p	3.81	3.08	1.56	0.03
hsa-miR-135b-5p	n. d.	-2.28	n. d.	
hsa-miR-141-3p	3.69	12.43	2.40	
hsa-miR-143-3p	4.95	n. d.	n. d.	
hsa-miR-145-5p	2.58	n. d.	-1.07	
hsa-miR-146a-5p	n. d.	n. d.	n. d.	
hsa-miR-146b-5p	n. d.	n. d.	n. d.	
hsa-miR-148a-3p	-4.28	-2.57	1.28	
hsa-miR-15a-5p	n. d.	-1.12	n. d.	
hsa-miR-15b-5p	2.22	-1.41	1.17	
hsa-miR-16-5p	2.77	1.35	1.24	
hsa-miR-17-5p	3.83	6.31	1.85	0.03
hsa-miR-17-3p	n. d.	n. d.	n. d.	
hsa-miR-181a-5p	n. d.	n. d.	n. d.	
hsa-miR-181b-5p	2.55	-4.35	1.93	
hsa-miR-182-5p	n. d.	-2.08	n. d.	
hsa-miR-183-5p	n. d.	-6.64	n. d.	
hsa-miR-184	n. d.	n. d.	n. d.	
hsa-miR-194-5p	3.24	-1.31	1.01	
hsa-miR-195-5p	1.15	1.88	1.65	
hsa-miR-196a-5p	n. d.	n. d.	n. d.	
hsa-miR-19b-3p	-1.46	1.74	3.57	
hsa-miR-200b-3p	2.06	2.10	1.01	
hsa-miR-200c-3p	1.90	11.15	-1.19	
hsa-miR-203a-3p	2.09	-2.39	-1.32	
hsa-miR-205-5p	2.91	-2.16	1.05	
hsa-miR-20a-5p	2.58	10.68	9.53	0.02
hsa-miR-20b-5p	2.62	4.98	2.65	0.03
hsa-miR-21-5p	-1.08	-3.73	-1.25	
hsa-miR-218-5p	n. d.	n. d.	n. d.	
hsa-miR-22-3p	-2.94	-1.26	-2.62	0.01
hsa-miR-221-3p	4.08	-2.01	1.47	
hsa-miR-222-3p	n. d.	-3.24	n. d.	
hsa-miR-223-3p	7.18	n. d.	2.01	
hsa-miR-224-5p	n. d.	n. d.	n. d.	
hsa-miR-23b-3p	4.32	2.03	-1.74	

hsa-miR-24-3p	-1.36	1.01	-1.41	
hsa-miR-25-3p	1.06	-1.63	1.53	
hsa-miR-26a-5p	1.23	11.13	2.14	
hsa-miR-26b-5p	1.07	7.92	1.39	
hsa-miR-27a-3p	-1.23	-1.77	-1.66	
hsa-miR-27b-3p	1.63	-1.77	-1.02	
hsa-miR-296-5p	n. d.	n. d.	n. d.	
hsa-miR-29b-3p	3.96	4.41	2.89	0.02
hsa-miR-30c-5p	2.68	7.63	-1.03	
hsa-miR-31-5p	n. d.	-4.90	n. d.	
hsa-miR-3163	n. d.	n. d.	n. d.	
hsa-miR-32-5p	n. d.	-4.10	n. d.	
hsa-miR-330-3p	n. d.	n. d.	n. d.	
hsa-miR-331-3p	n. d.	-1.37	n. d.	
hsa-miR-34a-5p	n. d.	-1.43	n. d.	
hsa-miR-34b-3p	n. d.	n. d.	n. d.	
hsa-miR-34c-5p	n. d.	n. d.	n. d.	
hsa-miR-361-5p	n. d.	-3.13	n. d.	
hsa-miR-365b-3p	-1.12	-1.08	2.65	
hsa-miR-375	3,85	1,84	1.65	
hsa-miR-3662	n. d.	n. d.	n. d.	
hsa-miR-3666	5.39	n. d.	1.91	
hsa-miR-374c-5p	n. d.	2.39	n. d.	
hsa-miR-425-5p	4.15	-1.38	1.50	
hsa-miR-449a	n. d.	n. d.	n. d.	
hsa-miR-455-5p	1.33	n. d.	-2.09	
hsa-miR-494-3p	n. d.	-1.80	n. d.	
hsa-miR-616-3p	n. d.	n. d.	n. d.	
hsa-miR-7-5p	n. d.	n. d.	n. d.	
hsa-miR-9-3p	n. d.	n. d.	n. d.	
hsa-miR-92a-3p	-1.66	1.61	1.18	
hsa-miR-93-5p	n. d.	2.80	1.59	
hsa-miR-96-5p	n. d.	-1.41	n. d.	
hsa-miR-99a-5p	2.22	8.86	7.25	0.04
hsa-miR-99b-5p	11.33	1.25	1.84	

Abbreviations and codes: CP/CPPS: chronic prostatitis chronic pelvic pain syndrome; IDs: identities; n. d.: not determinable; In red: **s**ignificantly upregulated miRNAs in CP/CPPS patients; In green: significantly downregulated miRNAs in CP/CPPS (Student's t-test, p-values <0.05 were considered as significant; technical replicates were used).

Supplementary Table 8 Gene ontology enrichment analysis on 825 putative target genes of miRNAs*, which were significantly upregulated in post-prostatic-massage (PPM) urine exosomes of CP/CPPS patients

Gene ontology ID	Description	adjusted p-value	Gene IDs
GO:0050767	Regulation of neurogenesis (n=37)	8.87E-04	*BHLHE41, BMPR2, CAPRIN2, DYNLT1, E2F1, EGR2, EIF4G2, EPHA4, EPHA7, FBXO31, FZD3, HIF1A, LDLR, LIF, LIMK1, LRP8, MYCN, NDEL1, PAFAH1B1, PARP6, PLAG1, PLXNA1, PTEN, PTPRD, RAPGEF2, REST, RGMA, RNF6, SEMA4B, SEMA7A, SHTN1, SKIL, SORL1, SS18L1, STK11, TIAM1, ULK1*
GO:0007265	Ras protein signal transduction (n=35)	8.87E-04	*ABCA1, AKAP13, ARHGAP1, ARHGAP35, ARHGEF10, ARHGEF11, ARHGEF28, ARHGEF3, CDKN1A, CRK, DYNLT1, GBF1, HEG1, IQSEC2, LIMK1, LRRD1, MFN2, PSD, PTH, RAB12, RAP2C, RAPGEF2, RASGEF1A, RB1, RHOV, SHOC2, SHTN1, SOS1, SQSTM1, SSX2IP, STARD8, TIAM1, TIMP2, TNFAIP1, USP28*
GO:0051056	Regulation of small GTPase mediated signal transduction (n=33)	8.87E-04	*ABCA1, AKAP13, ARHGAP1, ARHGAP12, ARHGAP26, ARHGAP35, ARHGEF10, ARHGEF11, ARHGEF28, ARHGEF3, CRK, DYNLT1, FAM13A, FGD1, FGD4, FGD5, GBF1, HEG1, IQSEC2, LRRD1, MFN2, PREX1, PSD, RASGEF1A, SHOC2, SOS1, SQSTM1, SSX2IP, STARD8, TAGAP, TIAM1, TIMP2, TNFAIP1*
GO:0031331	Positive regulation of cellular catabolic process (n=40)	1.54E-03	*AADAC, ADAM9, AGTPBP1, ANKIB1, APP, ATG16L1, ATG7, BCL2L11, BNIP3L, CNOT6L, CNOT7, CSNK1A1, DAB2, FBXL5, FURIN, FYCO1, HIF1A, HTR2A, ITCH, LDLR, MAPK9, MEX3D, NANOS1, ORMDL3, PAFAH1B2, PIP4K2A, PTEN, RGMA, RNF128, RNF217, SESN3, SMAD7, SPTLC1, STK11, TNRC6C, TP53INP1, TP53INP2, TRIM8, ULK1, YTHDF3*
GO:0043087	Regulation of GTPase activity (n=33)	3.98E-03	*ARAP2, ARHGAP12, ARHGEF10, CCL1, CRK, DYNLT1, EIF2S1, EPHA4, EPHA5, FGD1, FGD4, FGD5, FICD, GNB5, GPR137B, LRCH1, NDEL1, OCRL, PAFAH1B1, PLXNA1, PREX1, RAPGEF2, RGMA, RUNDC1, S1PR1, TBC1D15, TBC1D17, TBC1D2, TBC1D20, TBC1D8B, TBC1D9, TIAM1, USP6*

Codes: * In total nine miRNAs were significantly upregulated in PPM urine exosomes of CP/CPPS patients in comparison to healthy men, and genes being common targets of at least four upregulated miRNAs were considered for GO enrichment analysis (n=825)

Supplementary Table 9 Hallmark gene sets enrichment analysis using Molecular Signature Database on 825 putative target genes of miRNAs*, which were significantly upregulated in post-prostatic-massage (PPM) urine exosomes of CP/CPPS patients

**Hallmark_Description**	**adjusted p-value**	**Gene ID**
HALLMARK_APOPTOSIS (n=21)	1.78E-03	*APP, BCL2L11, BNIP3L, BTG3, CASP8, CCND1, CCND2, CD69, CDKN1A, ERBB3, EREG, ETF1, IRF1, MCL1, MMP2, PPP3R1, SMAD7, SQSTM1, TIMP2, TXNIP, WEE1*
HALLMARK_G2M_CHECKPOINT (n=20)	3.32E-02	*ARID4A, CCND1, CHAF1A, E2F1, E2F2, HIF1A, HMGB3, HSPA8, JPT1, KIF23, KPNA2, PAFAH1B1, PBK, POLQ, RAD23B, RBL1, RPA2, RPS6KA5, SRSF2, SYNCRIP*
HALLMARK_IL2_STAT5_SIGNALING (n=21)	1.68E-02	*AHNAK, ARL4A, BMPR2, CCND2, FAM126B, FGL2, FURIN, IKZF4, LIF, LRIG1, MAP3K8, NCOA3, P2RX4, PHTF2, RNH1, RORA, RRAGD, SLC29A2, TIAM1, TNFRSF21, TNFSF11*
HALLMARK_TNFA_SIGNALING_VIA_NFKB (n=25)	1.48E-03	*ABCA1, BTG3, CCND1, CD69, CDKN1A, CXCL6, DUSP2, EGR2, F3, FJX1, IRF1, KDM6B, KLF10, KLF9, LDLR, LIF, MAP3K8, MCL1, NFAT5, NR4A2, NR4A3, SIK1, SLC16A6, SQSTM1, TRIP10*
HALLMARK_UV_RESPONSE_DN (n=18)	6.77E-03	*AKT3, APBB2, ATXN1, CELF2, DAB2, F3, HAS2, KCNMA1, LDLR, MIOS, PTEN, PTPN21, RUNX1, SFMBT1, SMAD7, TGFBR2, VLDLR, WDR37*

Codes: * In total nine miRNAs were significantly upregulated in PPM urine exosomes of CP/CPPS patients in comparison to healthy men, and genes being common targets of at least four upregulated miRNAs were considered for Hallmark gene sets enrichment analysis (n=825)

Supplementary Table 10 Kyoto Encyclopedia of Genes and Genomes (KEGG) pathway enrichment analysis on 825 putative target genes of miRNAs*, which were significantly upregulated in post-prostatic-massage (PPM) urine exosomes of CP/CPPS patients

KEGG pathway ID	Description	adjusted p-value	Gene IDs
hsa04144	Endocytosis (n=27)	1.71E-03	*AP2B1, ARAP2, DAB2, EEA1, GBF1, HSPA8, IQSEC2, ITCH, KIF5A, LDLR, LDLRAP1, NEDD4L, PARD6B, PDGFRA, PSD, RAB10, RAB11FIP1, RAB11FIP5, RAB22A, RAB5B, RABEP1, RUFY2, SNX12, TGFBR2, TSG101, VPS26A, ZFYVE9*
hsa04010	MAPK signaling pathway (n=29)	2.11E-03	*AKT3, CRK, DUSP2, DUSP8, ELK4, ERBB3, EREG, FGF5, FLT1, HSPA8, MAP3K1, MAP3K11, MAP3K12, MAP3K14, MAP3K2, MAP3K3, MAP3K5, MAP3K8, MAPK9, MKNK2, PDGFRA, PPP3R1, RAPGEF2, RPS6KA5, SOS1, TAOK1, TAOK2, TAOK3, TGFBR2*
hsa05215	Prostate cancer (n=13)	6.89E-03	*AKT3, CCND1, CDKN1A, CREB5, E2F1, E2F2, MMP3, PDGFRA, PIK3R1, PTEN, RB1, SOS1, TCF7L1*
hsa04068	FoxO signaling pathway (n=16)	6.89E-03	*AKT3, BCL2L11, CCND1, CCND2, CCNG2, CDKN1A, MAPK9, PIK3R1, PTEN, RBL2, S1PR1, SLC2A4, SOS1, STAT3, STK11, TGFBR2*
hsa05223	Non-small cell lung cancer (n=11)	6.89E-03	*AKT3, CCND1, CDKN1A, E2F1, E2F2, KIF5A, PIK3R1, RARB, RB1, SOS1, STAT3*

Codes: * In total nine miRNAs were significantly upregulated in PPM urine exosomes of CP/CPPS patients in comparison to healthy men, and genes being common targets of at least four upregulated miRNAs were considered for KEGG pathway enrichment analysis (n=825)

Supplementary Table 11 RT² Profiler™ PCR Array „Human Cancer Inflammation & Immunity Crosstalk" in untreated THP-1 (M0) cells and THP-1 treated for 24 hours with post-prostatic-massage (PPM) urine exosomes of healthy men (n=2) and CP/CPPS patients (n=3)

RT² array Position	Gene Symbol	Gene Description	Healthy		CP/CPPS	
			#Fold regulation	Note	#Fold regulation	Note
A01	*ACKR3*	Chemokine (C-X-C motif) receptor 7	1.48		-1.95	
A02	***AICDA****	Activation-induced cytidine deaminase	-3.94	C	-3.39	C
A03	*BCL2*	B-cell CLL/lymphoma 2	-1.19	A	1.02	A
A04	*BCL2L1*	BCL2-like 1	1.01	A	-1.70	
A05	*CCL18*	Chemokine (C-C motif) ligand 18 (pulmonary and activation-regulated)	-1.45		3.83	
A06	*CCL2*	Chemokine (C-C motif) ligand 2	1.23		-1.72	
A07	*CCL20*	Chemokine (C-C motif) ligand 20	1.33	A	1.08	
A08	*CCL21*	Chemokine (C-C motif) ligand 21	-2.82		1.20	
A09	*CCL22*	Chemokine (C-C motif) ligand 22	1.71		-2.02	
A10	*CCL28*	Chemokine (C-C motif) ligand 28	1.12	A	2.10	A
A11	*CCL4*	Chemokine (C-C motif) ligand 4	-2.31		-4.78	
A12	*CCL5*	Chemokine (C-C motif) ligand 5	-1.72		-2.39	
B01	*CCR1*	Chemokine (C-C motif) receptor 1	1.26	A	1.25	A
B02	*CCR10*	Chemokine (C-C motif) receptor 10	-2.58		-1.09	
B03	*CCR2*	Chemokine (C-C motif) receptor 2	-1.35		6.79	A
B04	*CCR4*	Chemokine (C-C motif) receptor 4	-4.62		-2.38	
B05	*CCR7*	Chemokine (C-C motif) receptor 7	-3.94	C	-3.39	C
B06	*CCR9*	Chemokine (C-C motif) receptor 9	-1.23		-1.42	
B07	***CD274****	CD274 molecule	-1.68		1.21	
B08	***CSF1****	Colony stimulating factor 1 (macrophage)	-1.02		-8.01	A
B09	*CSF2*	Colony stimulating factor 2 (granulocyte-macrophage)	-1.16		1.20	
B10	*CSF3*	Colony stimulating factor 3 (granulocyte)	-6.66		-4.12	
B11	*CTLA4*	Cytotoxic T-lymphocyte-associated protein 4	-3.94	C	-3.39	C
B12	*CXCL1*	Chemokine (C-X-C motif) ligand 1 (melanoma growth stimulating activity, alpha)	-1.65		1.29	
C01	***CXCL10****	Chemokine (C-X-C motif) ligand 10	-4.92		-2.51	
C02	*CXCL11*	Chemokine (C-X-C motif) ligand 11	-3.94	C	-3.39	C
C03	***CXCL12****	Chemokine (C-X-C motif) ligand 12	-3.23		-1.91	
C04	*CXCL2*	Chemokine (C-X-C motif) ligand 2	-2.01		-1.05	
C05	*CXCL5*	Chemokine (C-X-C motif) ligand 5	-1.78		-1.59	
C06	*CXCL9*	Chemokine (C-X-C motif) ligand 9	-3.86		-1.32	
C07	*CXCR1*	Chemokine (C-X-C motif) receptor 1	-3.94	C	-2.01	
C08	*CXCR2*	Chemokine (C-X-C motif) receptor 2	-2.23	A	-1.21	A
C09	*CXCR3*	Chemokine (C-X-C motif) receptor 3	-2.39		-1.97	
C10	*CXCR4*	Chemokine (C-X-C motif) receptor 4	1.21		1.76	
C11	*CXCR5*	Chemokine (C-X-C motif) receptor 5	-3.29		-2.32	
C12	*EGF*	Epidermal growth factor	-5.01		-2.47	
D01	*EGFR*	Epidermal growth factor receptor	-3.94	C	-3.39	C
D02	*FASLG*	Fas ligand (TNF superfamily, member 6)	-3.48		-1.58	
D03	*FOXP3*	Forkhead box P3	-3.34		-2.20	A

D04	*GBP1*	Guanylate binding protein 1, interferon-inducible	1.06	A	-1.28	
D05	*GZMA*	Granzyme A (granzyme 1, cytotoxic T-lymphocyte-associated serine esterase 3)	-3.84		-2.76	
D06	*GZMB*	Granzyme B (granzyme 2, cytotoxic T-lymphocyte-associated serine esterase 1)	-3.93		-2.18	
D07	*HIF1A*	Hypoxia inducible factor 1, alpha subunit (basic helix-loop-helix transcription factor)	-1.06		-2.08	
D08	*HLA-A*	Major histocompatibility complex, class I, A	1.94		2.31	
D09	*HLA-B*	Major histocompatibility complex, class I, B	-1.48		-1.32	
D10	*HLA-C*	Major histocompatibility complex, class I, C	-3.55		-2.39	
D11	*IDO1*	Indoleamine 2,3-dioxygenase 1	-3.94	C	-3.39	C
D12	***IFNG****	Interferon, gamma	-3.94	C	-2.75	
E01	***IGF1****	Insulin-like growth factor 1 (somatomedin C)	-3.47		-2.20	
E02	*IL10*	Interleukin 10	-2.24		-2.18	
E03	*IL12A*	Interleukin 12A (natural killer cell stimulatory factor 1, cytotoxic lymphocyte maturation factor 1, p35)	-2.91		1.18	
E04	*IL12B*	Interleukin 12B (natural killer cell stimulatory factor 2, cytotoxic lymphocyte maturation factor 2, p40)	-1.76		-2.18	
E05	*IL13*	Interleukin 13	-2.43		-5.82	
E06	*IL15*	Interleukin 15	-3.94	C	-3.39	C
E07	*IL17A*	Interleukin 17A	-3.94	C	-3.39	C
E08	*IL1A*	Interleukin 1, alpha	-1.71		-2.27	
E09	*IL1B*	Interleukin 1, beta	-1.24		-6.85	A
E10	*IL2*	Interleukin 2	-3.94	C	-3.39	C
E11	*IL23A*	Interleukin 23, alpha subunit p19	-1.00		1.89	A
E12	*IL4*	Interleukin 4	-3.94	C	-2.63	
F01	*IL6*	Interleukin 6 (interferon, beta 2)	-3.94	C	-2.38	
F02	***CXCL8****	Interleukin 8	-2.36		-3.15	
F03	***IRF1****	Interferon regulatory factor 1	1.39	A	1.77	A
F04	*KITLG*	KIT ligand	-2.34		-3.37	
F05	*MICA*	MHC class I polypeptide-related sequence A	-2.04		1.07	
F06	*MICB*	MHC class I polypeptide-related sequence B	-1.14		-1.33	
F07	*MIF*	Macrophage migration inhibitory factor (glycosylation-inhibiting factor)	-1.43		-1.24	
F08	***MYC****	V-myc myelocytomatosis viral oncogene homolog (avian)	1.15		2.35	
F09	*MYD88*	Myeloid differentiation primary response gene (88)	-1.57		1.09	
F10	*NFKB1*	Nuclear factor of kappa light polypeptide gene enhancer in B-cells 1	1.20	A	1.06	A
F11	*NOS2*	Nitric oxide synthase 2, inducible	-1.82		-2.05	
F12	*PDCD1*	Programmed cell death 1	-2.90		-3.52	
G01	*PTGS2*	Prostaglandin-endoperoxide synthase 2 (prostaglandin G/H synthase and cyclooxygenase)	-1.07		-1.90	
G02	*SPP1*	Secreted phosphoprotein 1	-1.01		-8.12	
G03	*STAT1*	Signal transducer and activator of transcription 1, 91kDa	-1.04		1.22	
G04	*STAT3*	Signal transducer and activator of transcription 3 (acute-phase response factor)	-1.00		1.01	
G05	*TGFB1*	Transforming growth factor, beta 1	1.13		1.21	
G06	*TLR2*	Toll-like receptor 2	1.23		2.29	

G07	*TLR3*	Toll-like receptor 3	-3.94	C	-3.22	
G08	*TLR4*	Toll-like receptor 4	1.61	A	1.84	A
G09	*TNF*	Tumor necrosis factor	-1.28		-1.63	
G10	*TNFSF10*	Tumor necrosis factor (ligand) superfamily, member 10	-1.39	A	1.28	A
G11	*TP53*	Tumor protein p53	1.26	A	1.49	A
G12	***VEGFA****	Vascular endothelial growth factor A	-1.27		1.01	
H01	*ACTB*	Actin, beta	1.33		-1.27	
H02	*B2M*	Beta-2-microglobulin	-1.12		1.11	
H03	*GAPDH*	Glyceraldehyde-3-phosphate dehydrogenase	1.51		1.27	
H04	*HPRT1*	Hypoxanthine phosphoribosyltransferase 1	-1.25	A	-1.04	A
H05	*RPLP0*	Ribosomal protein, large, P0	-1.43		-1.07	
H06	HGDC	Human Genomic DNA Contamination	n.a.	passed	n.a.	passed
H07	RTC	Reverse Transcription Control	n.a.	passed	n.a.	passed
H08	RTC	Reverse Transcription Control	n.a.	passed	n.a.	passed
H09	RTC	Reverse Transcription Control	n.a.	passed	n.a.	passed
H10	PPC	Positive PCR Control	n.a.	passed	n.a.	passed
H11	PPC	Positive PCR Control	n.a.	passed	n.a.	passed
H12	PPC	Positive PCR Control	n.a.	passed	n.a.	passed

Abbreviations and codes: #Fold regulation of genes was calculated in comparison to gene expression levels in untreated THP-1 cells at M0 state (PCR arrays were done in duplicates per each sample); *Predicted target genes of at least one out of nine significantly upregulated microRNAs in PPM urine of CP/CPPS patients; In green: > 2 fold down-regulation; In red: > 2 fold upregulation; n.a.: not applicable; Notes (Qiagen): A) This gene's average threshold cycle is relatively high (> 30) in either the control or the test sample, and is reasonably low in the other sample (< 30). These data mean that the gene's expression is relatively low in one sample and reasonably detected in the other sample suggesting that the actual fold-change value is at least as large as the calculated and reported fold-change result; B) This gene's average threshold cycle is relatively high (> 30), meaning that its relative expression level is low, in both control and test samples, and the p-value for the fold-change is either unavailable or relatively high (p > 0.05) (case B did not occur in our results); C) This gene's average threshold cycle is either not determined or greater than the defined cut-off (default 35), in both samples meaning that its expression was undetected, making this fold-change result erroneous and uninterpretable (case C was excluded from further analyses); Passed: Successful check oft he iIntegrated controls (human genomic DNA contamination, Reverse Transcription and PCR controls)

Supplementary Table 12 RT² Profiler™ PCR Array „Human Transcription Factors“ in untreated THP-1 (M0) cells and THP-1 treated for 24 hours with post-prostatic massage (PPM) urine exosomes of healthy men (n=2) and CP/CPPS patients (n=3)

RT² array Position	Gene symbol	Gene description	Healthy		CP/CPPS	
			#Fold regulation	Note	#Fold regulation	Note
A01	*AR*	Androgen receptor	1.04		2.22	A
A02	***ARNT****	Aryl hydrocarbon receptor nuclear translocator	1.12		1.54	
A03	*ATF1*	Activating transcription factor 1	-1.37		-1.45	
A04	*ATF2*	Activating transcription factor 2	-1.22		-1.02	
A05	***ATF3****	Activating transcription factor 3	1.01		-3.33	
A06	*ATF4*	Activating transcription factor 4 (tax-responsive enhancer element B67)	-1.08		1.41	
A07	*CEBPA*	CCAAT/enhancer binding protein (C/EBP), alpha	1.20		3.42	
A08	*CEBPB*	CCAAT/enhancer binding protein (C/EBP), beta	1.35	A	3.42	A
A09	*CEBPG*	CCAAT/enhancer binding protein (C/EBP), gamma	1.43		1.79	
A10	*CREB1*	CAMP responsive element binding protein 1	1.15		-15.43	A
A11	*CREBBP*	CREB binding protein	1.06		1.62	
A12	*CTNNB1*	Catenin (cadherin-associated protein), beta 1, 88kDa	-1.03		-1.51	
B01	*DR1*	Down-regulator of transcription 1, TBP-binding (negative cofactor 2)	1.03		-1.50	
B02	***E2F1****	E2F transcription factor 1	1.11		2.73	A
B03	*E2F6*	E2F transcription factor 6	1.10		1.10	
B04	*EGR1*	Early growth response 1	-1.61		-1.14	
B05	*ELK1*	ELK1, member of ETS oncogene family	-1.22		1.88	
B06	*ESR1*	Estrogen receptor 1	1.67		3.15	
B07	*ETS1*	V-ets erythroblastosis virus E26 oncogene homolog 1 (avian)	1.05		-1.67	
B08	*ETS2*	V-Ets erythroblastosis virus E26 oncogene homolog 2 (avian)	-1.32		1.55	
B09	***FOS****	FBJ murine osteosarcoma viral oncogene homolog	-1.06		1.05	
B10	*FOXA2*	Forkhead box A2	-1.31		-1.48	
B11	*FOXG1*	Forkhead box G1	1.86		1.15	
B12	***FOXO1****	Forkhead box O1	1.21		1.00	
C01	*GATA1*	GATA binding protein 1 (globin transcription factor 1)	-2.23		-1.43	
C02	*GATA2*	GATA binding protein 2	-1.13		2.62	
C03	***GATA3****	GATA binding protein 3	-1.43		1.03	
C04	*GTF2B*	General transcription factor IIB	-1.15		-1.26	
C05	*GTF2F1*	General transcription factor IIF, polypeptide 1, 74kDa	-1.16		1.17	
C06	*HAND1*	Heart and neural crest derivatives expressed 1	-1.89	C	-1.58	C
C07	*HAND2*	Heart and neural crest derivatives expressed 2	-1.89	C	-1.58	C
C08	*HDAC1*	Histone deacetylase 1	-1.10		1.36	
C09	***HIF1A****	Hypoxia inducible factor 1, alpha subunit (basic helix-loop-helix transcription factor)	-1.16		-2.45	
C10	*HNF1A*	HNF1 homeobox A	-1.89	C	-1.58	C

C11	*HNF4A*	Hepatocyte nuclear factor 4, alpha	-1.84		1.27	
C12	*HOXA5*	Homeobox A5	1.38		5.45	
D01	*HSF1*	Heat shock transcription factor 1	-1.19		1.82	
D02	***ID1****	Inhibitor of DNA binding 1, dominant negative helix-loop-helix protein	1.66		3.65	
D03	***IRF1****	Interferon regulatory factor 1	1.30	A	2.27	A
D04	***JUN****	Jun proto-oncogene	1.55	A	1.92	A
D05	*JUNB*	Jun B proto-oncogene	1.45		4.77	A
D06	*JUND*	Jun D proto-oncogene	-1.14		-1.26	
D07	*MAX*	MYC associated factor X	-1.19		1.19	
D08	***MEF2A****	Myocyte enhancer factor 2A	1.06		-1.86	
D09	***MEF2C****	Myocyte enhancer factor 2C	1.02		-1.10	
D10	***MYB****	V-myb myeloblastosis viral oncogene homolog (avian)	-1.23		8.71	
D11	***MYC****	V-myc myelocytomatosis viral oncogene homolog (avian)	1.22		2.05	
D12	***MYF5****	Myogenic factor 5	-1.86		-1.33	
E01	*MYOD1*	Myogenic differentiation 1	-1.89	C	-1.58	C
E02	***NFAT5****	Nuclear factor of activated T-cells 5, tonicity-responsive	1.04		-1.22	
E03	*NFATC1*	Nuclear factor of activated T-cells, cytoplasmic, calcineurin-dependent 1	-1.01		-1.01	
E04	***NFATC2****	Nuclear factor of activated T-cells, cytoplasmic, calcineurin-dependent 2	-1.43		1.59	
E05	***NFATC3****	Nuclear factor of activated T-cells, cytoplasmic, calcineurin-dependent 3	-23.02	A	-1.10	
E06	*NFATC4*	Nuclear factor of activated T-cells, cytoplasmic, calcineurin-dependent 4	-1.57		3.96	
E07	*NFKB1*	Nuclear factor of kappa light polypeptide gene enhancer in B-cells 1	-1.25		-1.52	
E08	*NFYB*	Nuclear transcription factor Y, beta	-1.07	A	1.38	A
E09	***NR3C1****	Nuclear receptor subfamily 3, group C, member 1 (glucocorticoid receptor)	1.04		-1.60	
E10	*PAX6*	Paired box 6	-1.89	C	-1.58	C
E11	*POU2AF1*	POU class 2 associating factor 1	-2.22		-1.06	
E12	***PPARA****	Peroxisome proliferator-activated receptor alpha	-1.24		1.23	
F01	*PPARG*	Peroxisome proliferator-activated receptor gamma	1.15		-1.26	
F02	***RB1****	Retinoblastoma 1	-1.18		-1.57	
F03	*REL*	V-rel reticuloendotheliosis viral oncogene homolog (avian)	-1.07		-1.79	
F04	*RELA*	V-rel reticuloendotheliosis viral oncogene homolog A (avian)	1.02		1.61	
F05	*RELB*	V-rel reticuloendotheliosis viral oncogene homolog B	-1.97		-1.51	
F06	*SMAD1*	SMAD family member 1	-1.49		1.07	
F07	***SMAD4****	SMAD family member 4	-1.07		1.54	
F08	***SMAD5****	SMAD family member 5	-1.25		1.09	
F09	*SMAD9*	SMAD family member 9	-1.89	C	1.30	
F10	***SP1****	Sp1 transcription factor	1.34		1.72	
F11	***SP3****	Sp3 transcription factor	-1.02		1.00	
F12	*STAT1*	Signal transducer and activator of transcription 1, 91kDa	-1.08		-1.01	
G01	*STAT2*	Signal transducer and activator of transcription 2, 113kDa	-1.12		4.35	

G02	***STAT3****	Signal transducer and activator of transcription 3 (acute-phase response factor)	-1.01		-1.01	
G03	*STAT4*	Signal transducer and activator of transcription 4	-5.31		-5.70	
G04	*STAT5A*	Signal transducer and activator of transcription 5A	-1.12		1.85	
G05	*STAT5B*	Signal transducer and activator of transcription 5B	-1.07		1.55	
G06	***STAT6****	Signal transducer and activator of transcription 6, interleukin-4 induced	-1.01		1.34	
G07	*TBP*	TATA box binding protein	1.03	A	1.80	A
G08	***TCF7L2****	Transcription factor 7-like 2 (T-cell specific, HMG-box)	1.34		1.18	
G09	***TFAP2A****	Transcription factor AP-2 alpha (activating enhancer binding protein 2 alpha)	-1.05	A	1.36	A
G10	***TGIF1****	TGFB-induced factor homeobox 1	1.00		1.59	
G11	*TP53*	Tumor protein p53	1.03	A	2.26	A
G12	*YY1*	YY1 transcription factor	-1.07		1.21	
H01	*ACTB*	Actin, beta	1.10		1.08	
H02	*B2M*	Beta-2-microglobulin	1.06		1.09	
H03	*GAPDH*	Glyceraldehyde-3-phosphate dehydrogenase	2147.45	A	3365.25	A
H04	*HPRT1*	Hypoxanthine phosphoribosyltransferase 1	-1.18	A	-1.11	A
H05	*RPLP0*	Ribosomal protein, large, P0	1.01		-1.06	
H06	HGDC	Human Genomic DNA Contamination	n.a.	passed	n.a.	passed
H07	RTC	Reverse Transcription Control	n.a.	passed	n.a.	passed
H08	RTC	Reverse Transcription Control	n.a.	passed	n.a.	passed
H09	RTC	Reverse Transcription Control	n.a.	passed	n.a.	passed
H10	PPC	Positive PCR Control	n.a.	passed	n.a.	passed
H11	PPC	Positive PCR Control	n.a.	passed	n.a.	passed
H12	PPC	Positive PCR Control	n.a.	passed	n.a.	passed

Abbreviations and codes: #Fold regulation of genes was calculated in comparison to gene expression levels in untreated THP-1 cells at M0 state (PCR arrays were done in duplicates per each sample); *Predicted target genes of at least one out of nine significantly upregulated microRNAs in PPM urine of CP/CPPS patients; In green: > 2 fold down-regulation; In red: > 2 fold upregulation; n.a.: not applicable; Notes (Qiagen): A) This gene's average threshold cycle is relatively high (> 30) in either the control or the test sample, and is reasonably low in the other sample (< 30). These data mean that the gene's expression is relatively low in one sample and reasonably detected in the other sample suggesting that the actual fold-change value is at least as large as the calculated and reported fold-change result; B) This gene's average threshold cycle is relatively high (> 30), meaning that its relative expression level is low, in both control and test samples, and the p-value for the fold-change is either unavailable or relatively high (p > 0.05) (case B did not occur in our results); C) This gene's average threshold cycle is either not determined or greater than the defined cut-off (default 35), in both samples meaning that its expression was undetected, making this fold-change result erroneous and uninterpretable (case C was excluded from further analyses); Passed: Successful check oft he iIntegrated controls (human genomic DNA contamination, Reverse Transcription and PCR controls)
